# Non-neoplastic pathology at the crossroads between neck imaging and cardiothoracic imaging

**DOI:** 10.1186/s13244-019-0790-y

**Published:** 2019-12-04

**Authors:** Trinh T. Nguyen, Patricia E. Melendez, Katherine Kaproth-Joslin, Alok A. Bhatt

**Affiliations:** 1 University of Rochester Medical Center, 601 Elmwood Ave, Box 648, Rochester, NY 14642 USA; 2 Mayo Clinic, 4500 San Pablo Road, Jacksonville, FL 32224 USA

**Keywords:** Thoracic inlet, Head and neck imaging, Thoracic imaging, Radiology

## Abstract

The thoracic inlet is located at the crossroads between imaging of the neck and chest. It represents an important anatomic landmark, serving as the central conducting pathway for many vital structures extending from the neck into the chest and vice versa. Many important body systems are located within this region, including the enteric, respiratory, vascular, lymphatic, neurologic, and endocrine systems. A detailed examination of this region is essential when reviewing neck and thoracic imaging. This article will discuss the normal anatomic boundaries of the thoracic inlet and present an image-rich systematic discussion of the non-neoplastic pathology that can occur in this region. The neoplastic pathology of the thoracic inlet will be covered in a companion article.

## Key points

- The thoracic inlet is an important anatomic region from which various non-neoplastic pathologies can arise.
- It contains many vital body systems such as the enteric, respiratory, lymphatic, neurologic, musculoskeletal, and endocrine structures/systems, allowing for the development of a systematic approach to the review of the thoracic inlet.
- Many of these findings can be subtle and easily overlooked as the thoracic inlet is located at the crossroad between different imaging specialties.

## Introduction

The thoracic inlet is at the crossroads of imaging, often captured as the first or last set of images obtained during cross-sectional studies of the chest or neck respectively. This ultimately increases the chance this region is overlooked during the interpretation of the study, especially if both regions are imaged, as the neck radiologist may feel that it is part of the chest territory and the chest radiologist may feel that it is part of the neck territory. In addition, confident interpretation of this region can be difficult as many radiologists are fellowship trained in either neuroradiology or cardiothoracic imaging, but not both specialties.

When evaluating the thoracic inlet, it is important to tackle this region with a methodical plan in order to reduce the chance of missing clinically important findings. We propose a systematic approach to the imaging of the thoracic inlet based on the modified form of the classic medical school mnemonic “VINDICATE”, referring to the potential diagnostic etiologies that can be used to create a differential diagnosis (Table 1). The etiologies to consider in this region are vascular, infectious/inflammatory, neurologic (instead of neoplastic which will be covered in a separate article), degenerative, iatrogenic, congenital, autoimmune, traumatic, and endocrine etiologies. This article will discuss the normal anatomic boundaries of the thoracic inlet and present an image-rich systematic discussion of some of the non-neoplastic pathology that can occur in this region based on the mnemonic “VINDICATE”, using different imaging modalities, including plain film, computed tomography (CT), magnetic resonance (MR), and ultrasound (US) imaging. The neoplastic pathology of the thoracic inlet will be covered in a companion article.

**Table 1:** Representative non-neoplastic pathology of the thoracic inlet based on the mnemonic “VINDICATE”

Category	Examples
Vascular	Aberrant right subclavian artery, partial anomalous pulmonary venous return (PAPVR), internal jugular venous thrombosis, vasculitis
Infectious/inflammatory	Lemierre’s syndrome, mediastinitis, esophagitis
Neurologic	Traction injury of the brachial plexus/brachial plexopathy, perineural cyst/Tarlov cyst, laryngeal nerve injury
Degenerative	Esophageal diverticulum (Zenker’s, Killian-Jamieson), tracheal diverticulum, musculoskeletal degenerative changes (cervical osteophytosis, diffuse idiopathic skeletal hyperostosis (DISH), disc herniation
Iatrogenic	Tracheoesophageal fistula, radiation therapy, esophageal tear
Congenital	Tracheobronchomegaly, dilated thoracic duct, branchial cleft cyst, narrowed thoracic inlet, fibromatosis colli, aberrant right subclavian artery, PAPVR
Autoimmune	Hashimoto’s thyroiditis, systemic sclerosis causing esophageal dilatation, antiphospholipid syndrome causing thrombosis, thymic hyperplasia
Traumatic	Blunt or penetrating trauma causing injuries to the esophagus, trachea, vasculature, nerves, muscles, bones, and soft tissues. Examples include pneumomediastinum, sternocleidomastoid muscle hematoma
Endocrine	Thyroid goiter, parathyroid hyperplasia, Madelung disease

## Imaging modality

Multiple imaging modalities can be utilized to assess the thoracic inlet, including radiography, fluoroscopy, CT, MRI, and ultrasound. In general, radiography is limited in providing spatial details, however is an important screening tool in a trauma setting. Pneumomediastinum and subcutaneous emphysema are well detected by radiographs, as well as osseous abnormalities including conditions such as cervical osteophytes and diffuse idiopathic skeletal hyperostosis (DISH). Fluoroscopy is the workhorse in dynamic visualization of the esophagus. It is a helpful screening tool for esophageal abnormalities such as diverticula, ulcers, stenosis, dysmotility, and cancer. CT provides extensive spatial resolution and is the “gold standard” for evaluating most vascular pathology, cancer staging, and as a quick evaluation tool in the setting of trauma. Evaluation of superficial structures such as the thyroid gland, cervical lymph nodes, and cervical vascular structures can be easily performed with ultrasound, which has the added benefit of portability and lack of radiation exposure. MRI is an advanced modality, with superior soft tissue resolution, critical in the assessment of neural and lymphatic structures such as the brachial plexus and thoracic duct, which cannot be as reliably assessed with CT.

## Anatomy of the thoracic inlet

**Fig. 1:**
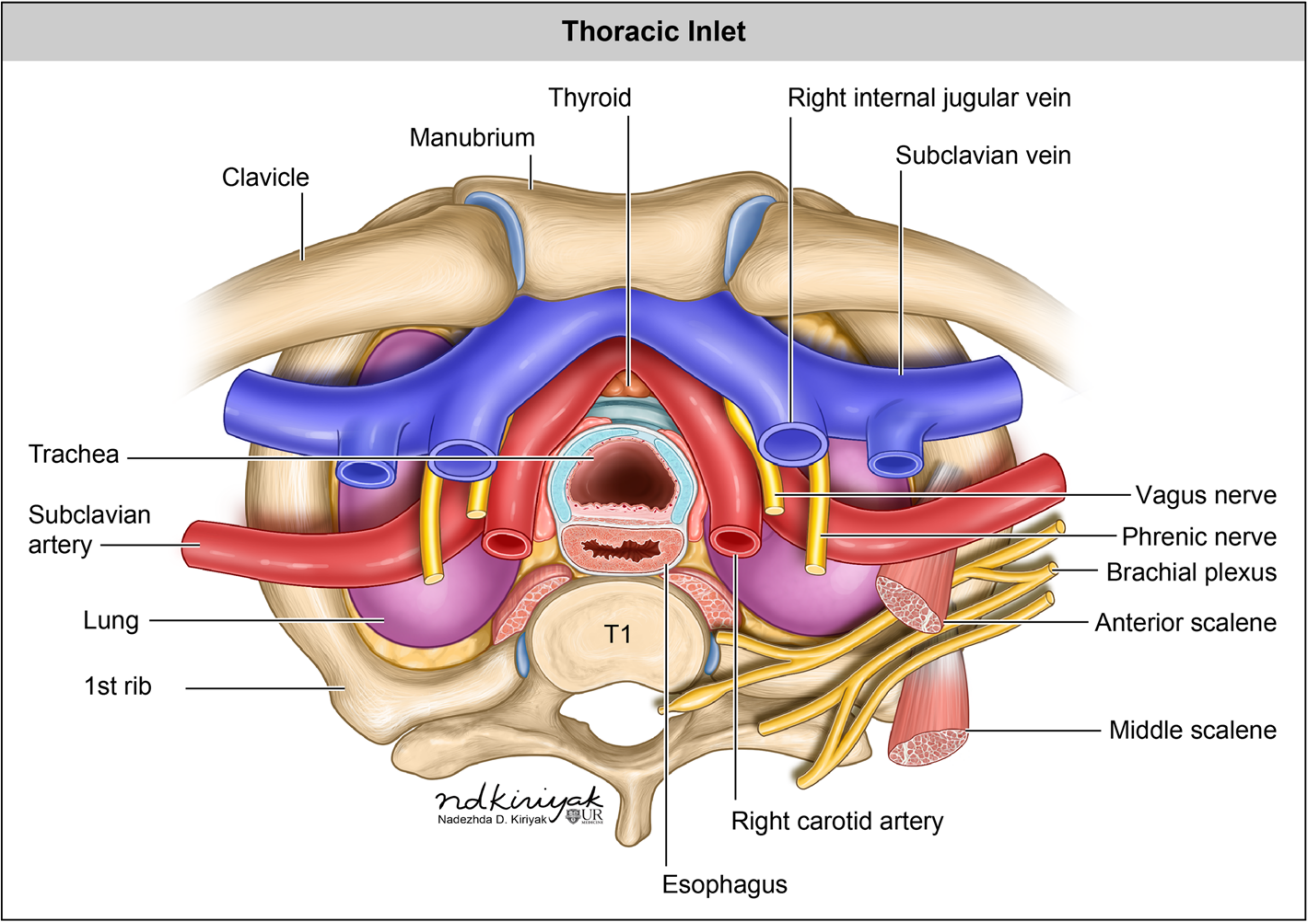
Anatomy of the thoracic inlet. The trachea is commonly located anterior and right lateral to the esophagus. The aortic arch typically gives rise to three main branches: the brachiocephalic artery (also known as the innominate artery or brachiocephalic trunk) which divides into the right common carotid artery and right subclavian, the left common carotid artery, and the left subclavian artery. The subclavian veins join the internal jugular veins to form the brachiocephalic veins (also known as innominate veins), which empty into the superior vena cava (SVC). The brachial plexus is comprised of nerve roots from cervical level 5 to thoracic level 1 and provides motor and sensory innervation to the shoulder and arm [1]. The phrenic nerve arises from cervical levels 3–5, running along the anterior surface of the anterior scalene muscle in the neck and enter the thorax posterior to the subclavian vein, providing innervation for the diaphragm. The bilateral vagus nerves are rarely directly discernible, even with high-resolution imaging. Their location, however, may be inferred by recognizing anatomic landmarks of their expected course and should be kept in mind during interpretation [1]. The thyroid isthmus lies just above the level of the thoracic inlet in the midline. The right and left lobes of the thyroid may extend inferiorly through the thoracic inlet into the mediastinum/substernal space [2]

The thoracic inlet is an important anatomic landmark as it contains multiple vital organ structures including the digestive tract, respiratory, vascular, lymphatic, endocrine, and neural structures. The boundaries of the thoracic inlet, however, are best defined by its surrounding osseous structures. The body of the first thoracic vertebrae makes up the posterior and superior margin of the thoracic inlet. The first pair of ribs and their costal cartilages define its lateral margins. Anteriorly and inferiorly, the thoracic inlet is bounded by the superior border of the manubrium. The inlet slopes anteroinferiorly following the first pair of ribs and spans roughly the first through third thoracic vertebral levels (Fig. 1) [1, 2].

Above the level of the first rib, familiarity with the cervical fasciae and infrahyoid neck spaces can help a radiologist construct a comprehensive differential diagnosis of pathology involving the thoracic inlet. The cervical fasciae define the spaces of the neck from which specific diseases can arise. They form important barriers which can limit the spread of infection and certain tumors [3].

**Fig. 2:**
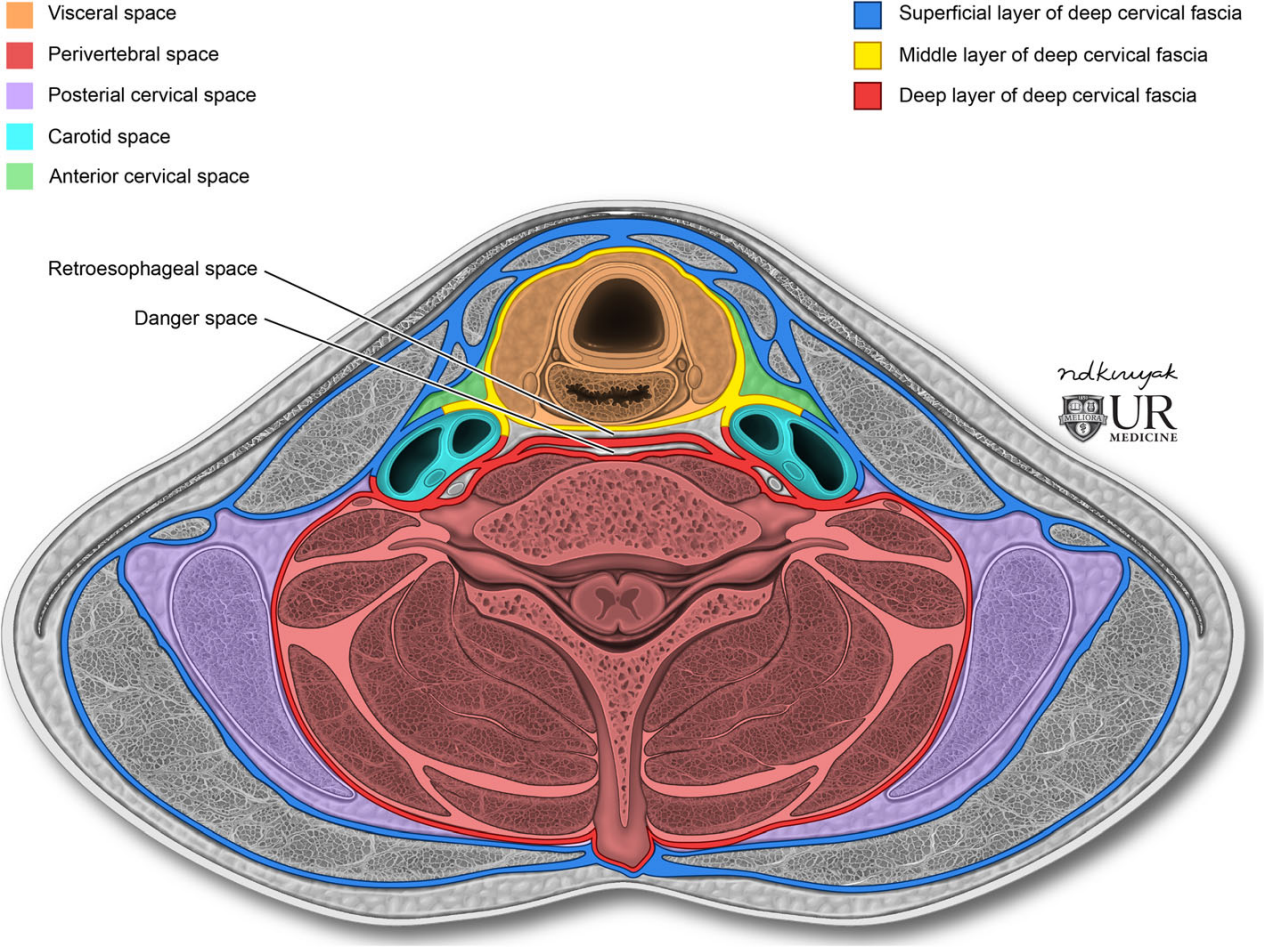
Infrahyoid deep neck spaces. The carotid space (CS) contains the cranial nerves IX–XII, internal jugular vein, and the internal carotid artery. The retropharyngeal space (RPS) is anterior to the vertebral column. The suprahyoid RPS contains fat and medial and lateral RPS nodes whereas the infrahyoid RPS contains fat only. The danger space (DS) is a potential space in the posterior RPS, separated from the anterior RPS by a facial “trap door”, approximately at the level of T3. RPS infection or tumor may spread to the mediastinum via this route. The visceral space (VS) contains thyroid and parathyroid glands, trachea, esophagus, recurrent laryngeal nerves, and pretracheal and paratracheal nodes. The posterior cervical space (PCS) contains fat, CNXI, and level V nodes. The perivertebral space (PVS) includes the prevertebral PVS and the paraspinal PVS. The prevertebral PVS contains brachial plexus and phrenic nerve, vertebral body, veins, arteries, and prevertebral and scalene muscles within. The paraspinal PVS contains only posterior vertebra elements and paraspinal muscles [3]

Traditionally, there are two major cervical fasciae, the superficial cervical fascia (SCF) and the deep cervical fascia (DCF). The SCF is a layer of fatty loose connective tissue that covers the head, face, and neck. Its primary function is to allow the skin to glide easily over the deeper structures of the neck. Infections that track along the SCF are superficial and rarely track deeper into the neck. The DCF is made up of denser layers which extend from the skull base inferiorly. There are three layers of the DCF: (1) the superficial layer of the deep cervical fascia (SLDCF) surrounds all the important neck structures; (2) the middle layer of the deep cervical fascia (MLDCF) includes the pretracheal and visceral layers which surround the aerodigestive tract; and (3) the deep layer of the deep cervical fascia (DLDCF) surrounds the vertebral column and paravertebral muscles. The three layers of the DCF delineate the infrahyoid neck spaces, from which we subdivide neck diseases. Figure 2 illustrates the anatomical contents of the infrahyoid neck spaces [3].

**Fig. 3:**
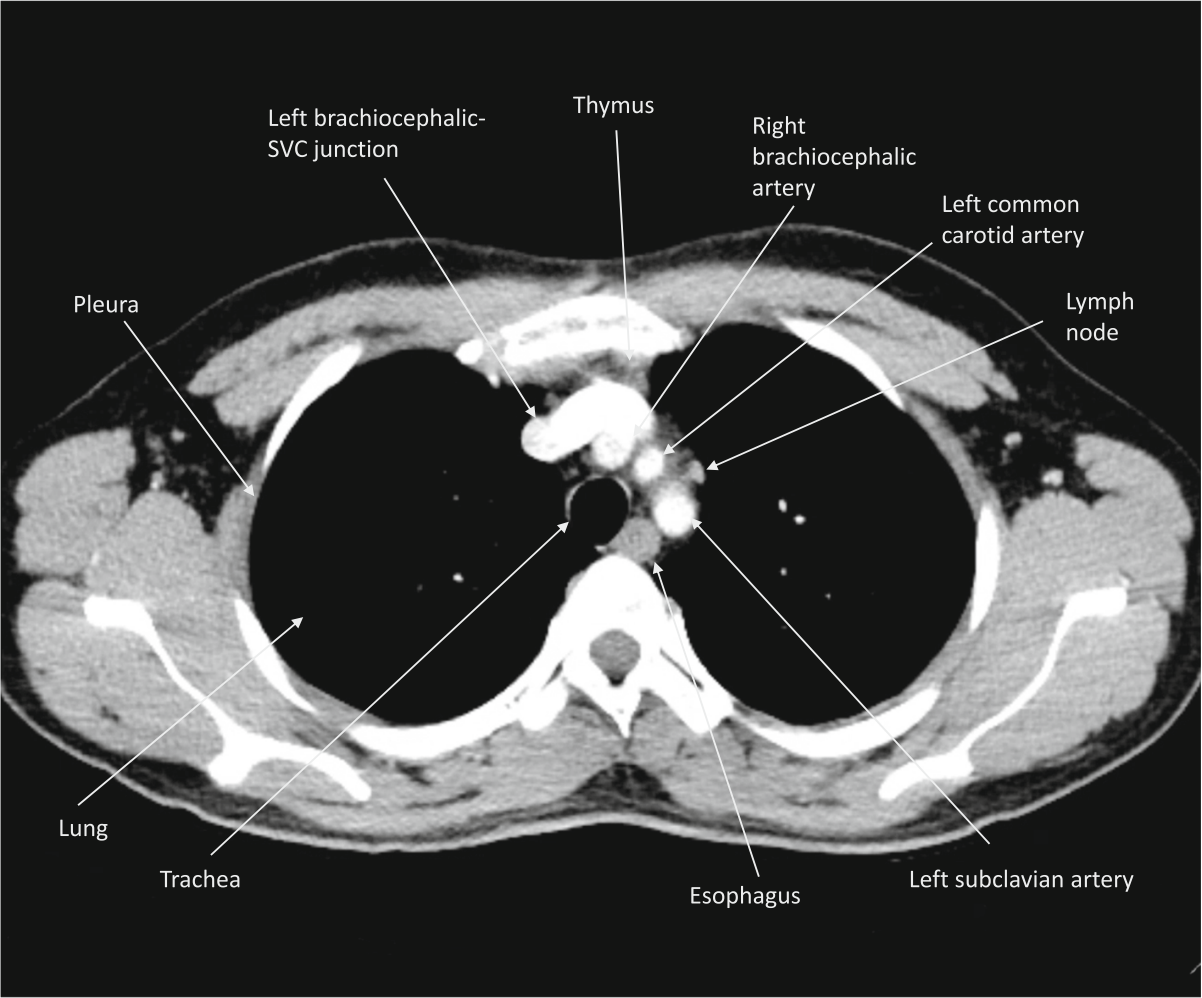
Anatomical contents of the upper thorax

Below the level of the first rib, familiarity with the structures of the upper thorax will play an important role in completing the differential diagnosis for the pathology of the thoracic inlet. The major structures of the upper thorax include the lungs, pleura/pleural space, mediastinum, vascular structures, and the chest wall. The mediastinum contains the esophagus, the trachea, the thymus, the lymph nodes, the thoracic duct, and multiple nerves including the phrenic nerve and the sympathetic chain. Figure 3 illustrates the anatomical contents of the upper thorax.

**Fig. 4:**
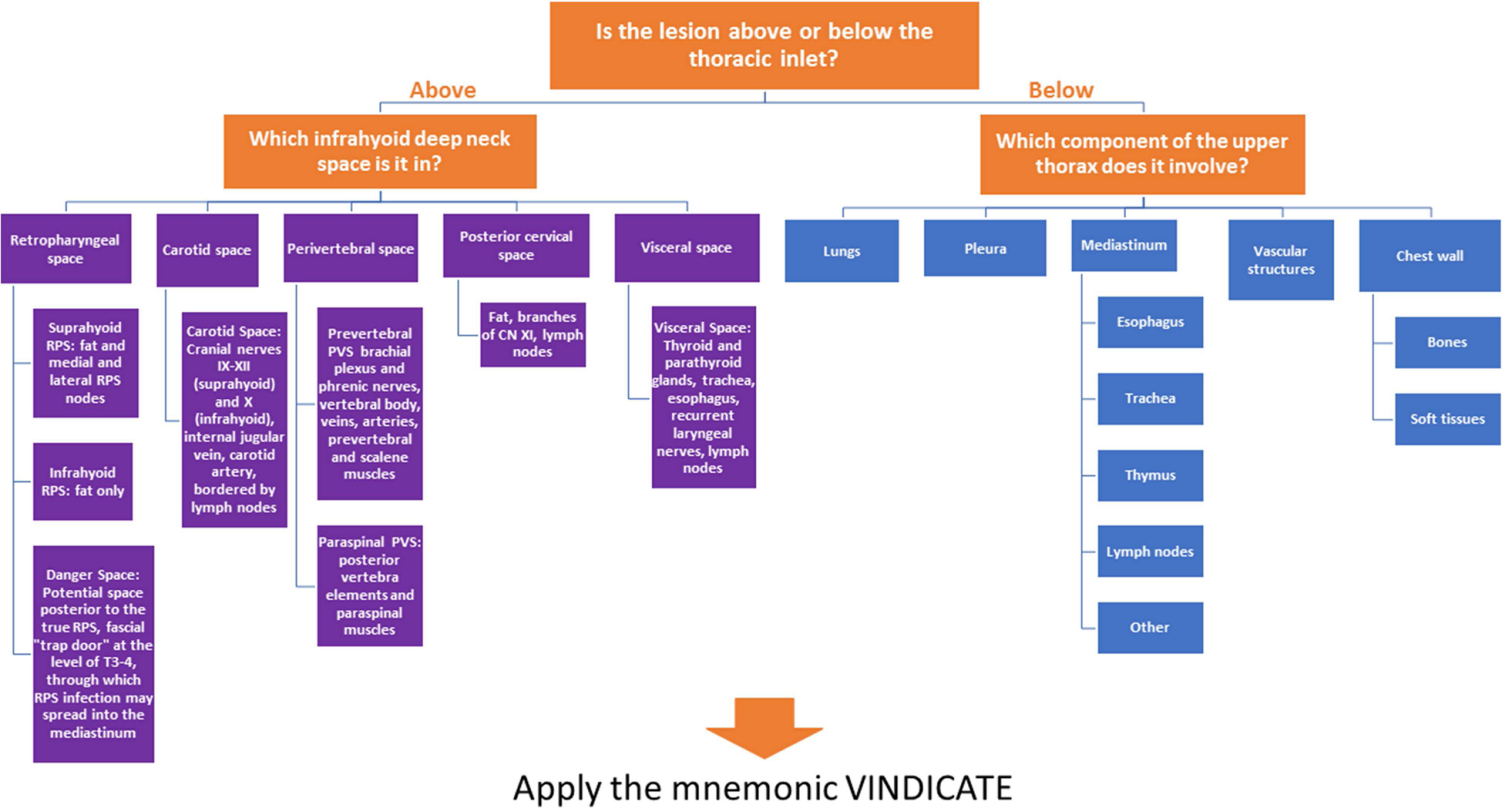
Algorithmic approach to pathology of the thoracic inlet region

Given the complex anatomy at the junction of the neck and thorax, an algorithmic approach should be used to determine which region of the thoracic inlet is affected (Fig. 4). Once the area of involvement is identified, applying the VINDICATE pneumonic to the region will help to expand the differential diagnosis to include a comprehensive list of possible causative etiologies.

## Vascular

Normal blood vessels in anomalous location and configuration can mimic a mass and be challenging to interpret. Familiarity with the more common vascular anomalies can increase confidence in interpretation. Alternatively, pathology involving the vasculature in the thoracic inlet can be easily overlooked.

### Aberrant right subclavian artery

**Fig. 5:**
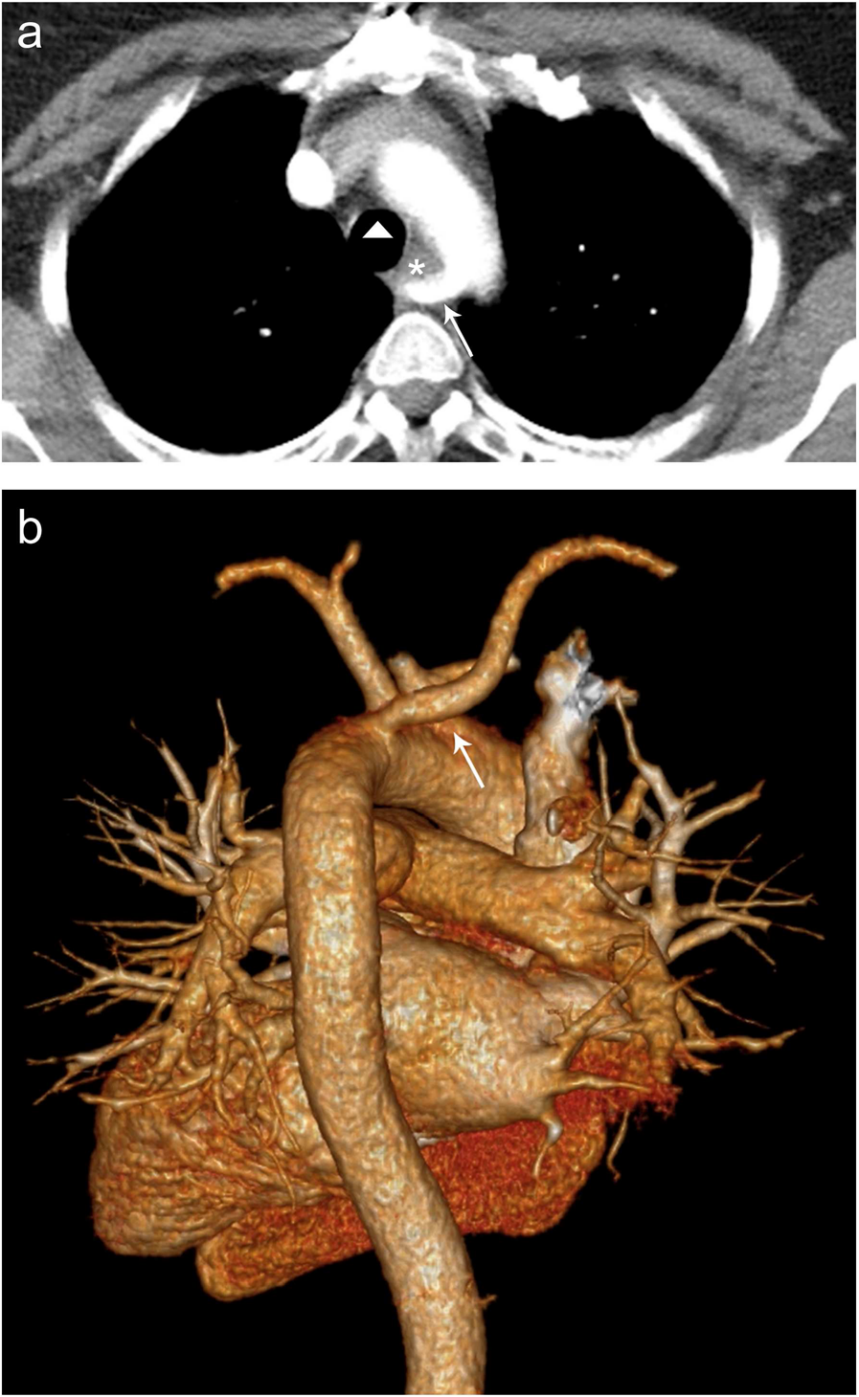
Aberrant right subclavian artery. Axial (**a**) CT image demonstrating aberrant right subclavian artery (arrow) coursing posterior to the esophagus (asterisk) and trachea (triangle). **b** 3D model demonstrating the distal take-off the aberrant right subclavian artery (arrow) from the aortic arch

Aberrant right subclavian artery is a developmental anomaly that is present in approximately 0.5–2% of the population and is the most common congenital anomaly of the aortic arch. It is caused by involution of the embryonic right 4th aortic arch between the left common carotid artery and left subclavian artery. Most patients are asymptomatic; however, symptoms may arise due to mass effect on the posterior esophagus causing dysphagia lusoria, and dyspnea and cough from posterior tracheal compression [4]. Radiography may demonstrate an ill-defined opacity in the right medial clavicular area located posterior to the trachea in the Raider triangle. An esophagram in the lateral view will show an oblique posterior impression on the esophagus. Both CT and MR can be easily used to demonstrate a right subclavian artery branch arising distal to the left subclavian artery origin from the aortic arch and coursing posterior to the trachea and esophagus (Fig. 5).

### Partial anomalous pulmonary venous return

**Fig. 6:**
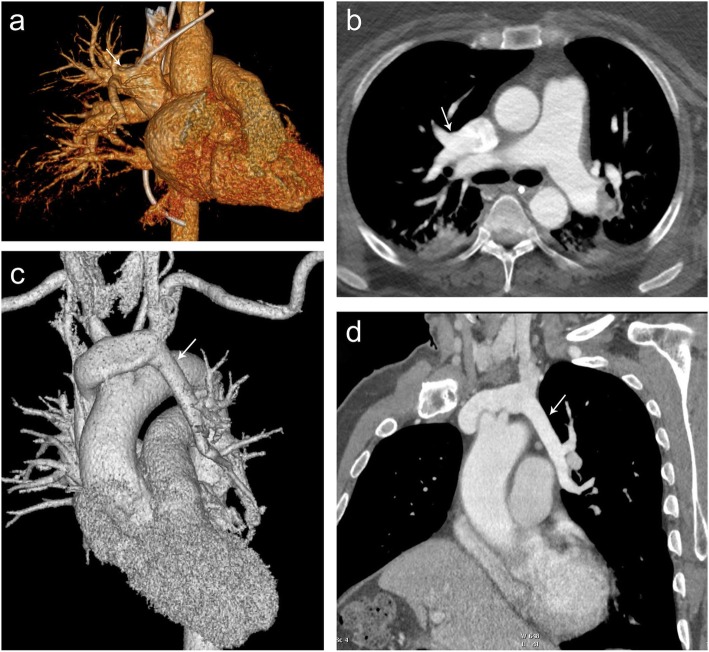
Partial anomalous pulmonary venous return. 3D reformat (**a**) and axial contrast-enhanced CT (**b**) demonstrating right upper lobe partial anomalous pulmonary venous return (arrow) draining into the SVC. 3D reformat (**c**) and double oblique (**d**) contrast-enhanced CT demonstrating left upper lobe partial anomalous pulmonary venous return (arrow) draining to the left brachiocephalic vein

Partial anomalous pulmonary venous return is a congenital anomaly in which the pulmonary vein drains into the systemic veins rather than into the left atrium (Fig. 6). Of the various types of partial anomalous pulmonary venous return (PAPVR), imaging at the level of the thoracic inlet may reveal abnormalities of the superior pulmonary veins. Symptoms, if present, typically are related to the left to right shunt and include dyspnea, palpitations, chest pain, tachycardia, edema, and systolic murmur. Right-sided PAPVR typically drains via the superior vena cava (SVC), and left-sided PAPVR typically drains via the left brachiocephalic vein. There may also be an associated sinus venosus atrial septal defect, more commonly present in the setting of a right PAPVR. MR imaging is equivalent to CT for morphologic characterization of PAPVR [5].

### Internal jugular vein thrombus

**Fig. 7:**
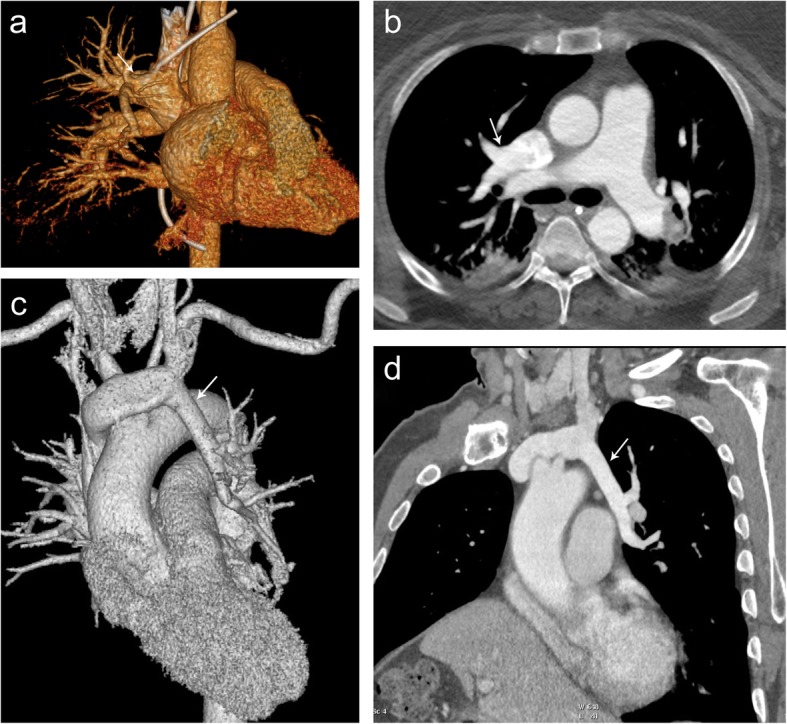
Internal jugular vein thrombus. Coronal contrast CT image demonstrating a catheter within the right internal jugular vein and nonopacification of the vessel, findings consistent with right internal jugular vein catheter-associated thrombus

**Fig. 8:**
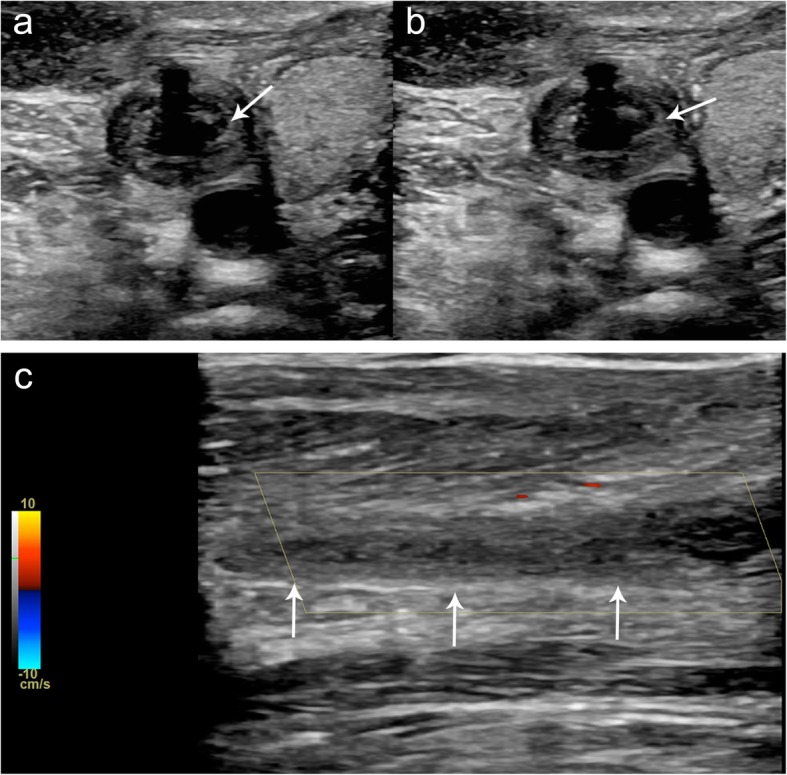
Internal jugular vein thrombus. Grayscale ultrasound images of the same patient in Fig. 7 with (**a**) and without compression (**b**) demonstrating heterogeneous echogenic material within the jugular vein which was noncompressible (arrow), findings consistent with thrombus. Note the catheter within the vessel. **c** Color Doppler ultrasound image demonstrate lack of blood flow (arrows) in the internal jugular vein confirming the presence of thrombus

Thrombosis of the internal jugular vein (IJV) arises from three main mechanisms: (1) endothelial damage from indwelling line or infection, which causes altered blood flow and hypercoagulable state; (2) venous stasis from compression of the neck (including internal jugular vein) or by compression of the mediastinal structures such as the SVC; and (3) malignancy as a risk factor for hypercoagulability. Trousseau syndrome refers to thromboembolism associated with malignancy, and many cancers such as pancreatic, lung, and ovarian cancers have been linked to this phenomenon [6]. Patients typically present with a swollen, hot, tender neck mass and fever in the acute phase (> 10 days) and a palpable tender cord in the neck in the chronic phase. Acute thrombus on CT imaging appears as a non-enhancing filling defect within a distended vein (Fig. 7). Occasionally, however, acute thrombus is hyperdense, best seen on noncontrast imaging, and can potentially mimic a patent IJV on venous (60–90 s delayed) phase of imaging; enlargement of the vein with circumferential fat stranding with adjacent edema in the retropharyngeal space can be clues to the diagnosis. A chronic thrombus typically appears as a well-marginated filling defect; linear stranding and calcifications may be present. The vessel size may be normal or small, and the associated adjacent soft tissue inflammatory changes have typically resolved. Collateral veins may be present. On ultrasound, the clot appears as a solid mass with intermediate echoes and the vein is non-compressible with absent flow on Doppler when the clot is occlusive (Fig. 8) [7].

## Infectious/inflammatory

Infection of the thoracic inlet region may arise spontaneously, as a result of traumatic injury, surgical complication, or extension from another space such as osteomyelitis or diskitis.

### Lemierre’s syndrome

**Fig. 9:**
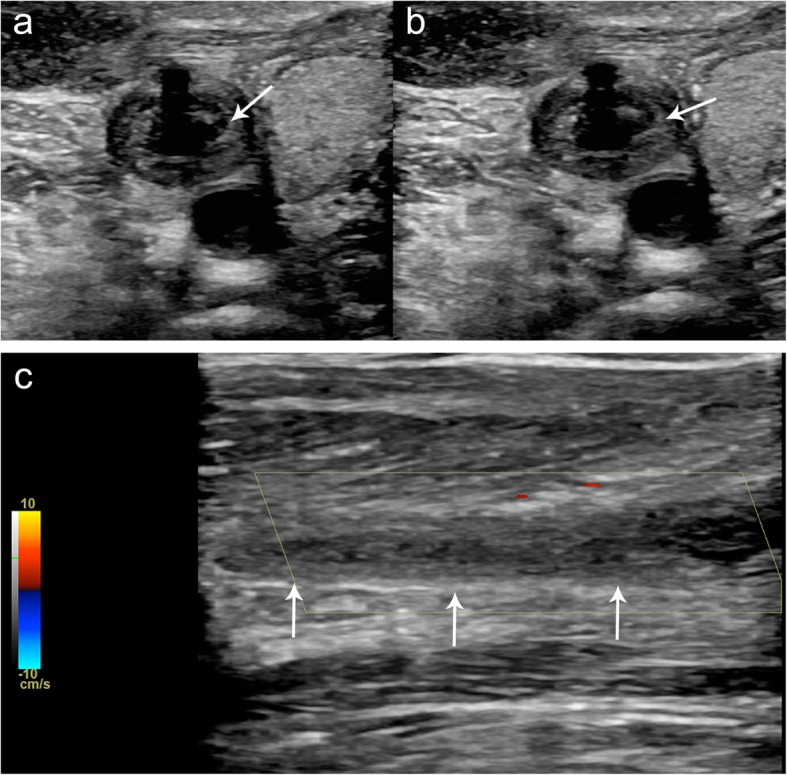
Lemierre’s syndrome. Contrast-enhanced axial (**a**) CT image of the thoracic inlet demonstrating a filling defect (asterisk) within the left internal jugular vein consistent with acute thrombus. Contrast-enhanced coronal (**b**) CT image demonstrates the extensive inflammatory changes and abscesses (arrow) within the soft tissues of the left neck. Together, these findings are consistent with the findings of Lemierre’s syndrome with extension into the thoracic inlet. Axial noncontrast CT (**c**) images in a companion case demonstrating multiple septic emboli within the pulmonary parenchyma (arrows). Note that a large right and small left complex pleural fluid collection (asterisk) was also present in this patient

Lemierre’s syndrome is a rare entity characterized by thrombophlebitis of the jugular veins secondary to upper aerodigestive tract infection, with or without peritonsillar or retropharyngeal abscess, otherwise known as post-pharyngitis venous thrombosis. It typically occurs in young adults and is caused by *Fusobacterium necrophorum* infection of the oropharynx in 80% of patients [8]. Patients present with ipsilateral tonsillar fullness and pain. The classic triad of finding includes neck vein thrombosis and cavitary pulmonary nodules in the setting of pharyngitis. CT imaging typically shows increased tonsil size, possible abscess, ipsilateral vein thrombosis (usually IJV), inflammatory changes of the neck and soft tissue edema, and metastatic seeding of infection (cavitary pulmonary nodules with the appearance of septic emboli, septic joints) [8] (Fig. 9).

### Mediastinitis

**Fig. 10:**
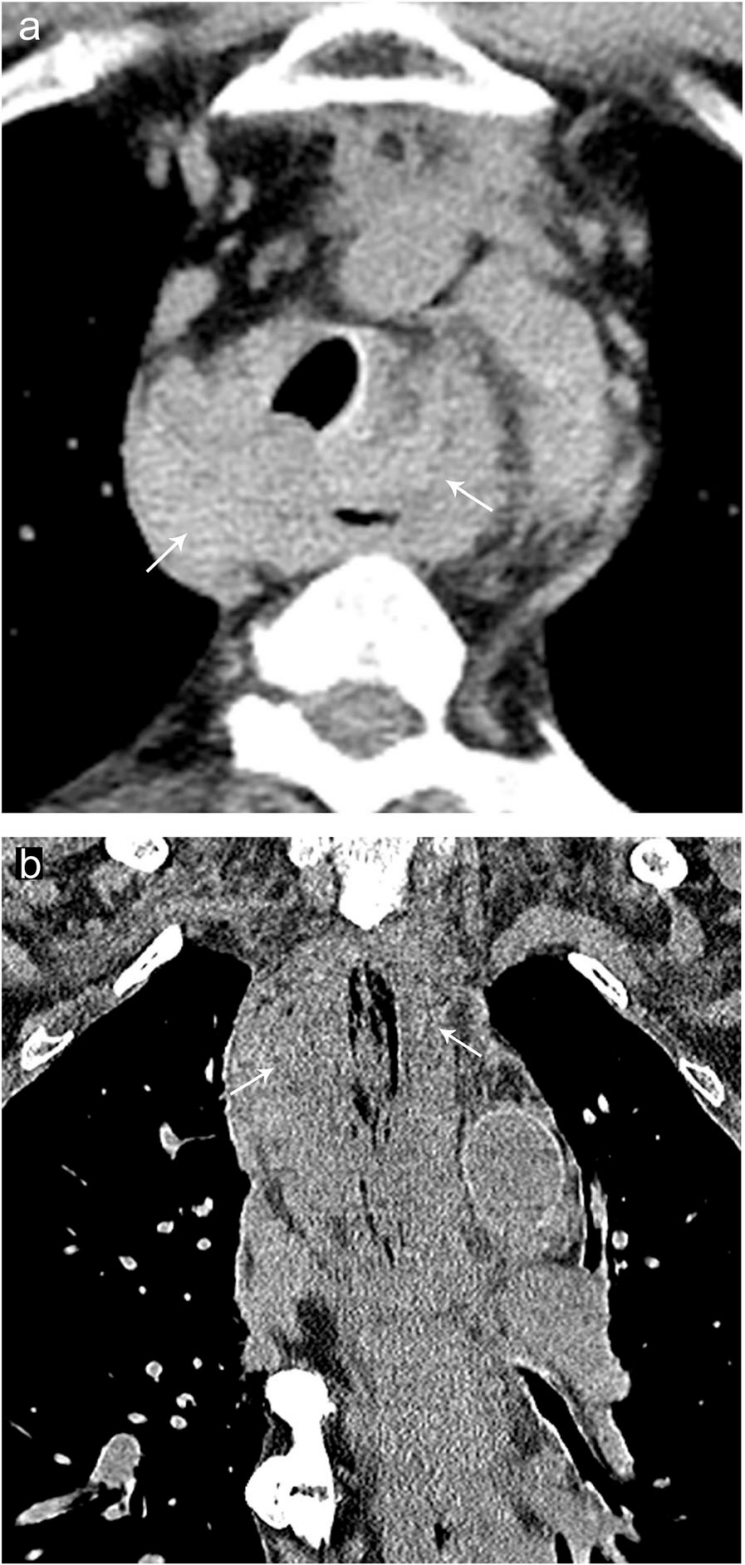
Mediastinitis. Axial (**a**) and coronal (**b**) noncontrast CT images demonstrating inflammatory changes and soft tissue stranding (arrows) of the mediastinum secondary to mediastinitis. Note the associated diffuse thickening of the esophageal wall in this patient with suspected esophageal wall perforation

Mediastinitis refers to the nonmalignant proliferation of fibrous tissue and acellular collagen in the mediastinum and is usually caused by an aggressive infectious or inflammatory process. While many cases of chronic mediastinitis are idiopathic, about 90% of cases of acute mediastinitis are secondary to esophageal perforation or rupture. Median sternotomy and radiation therapy are other important iatrogenic etiologies to consider. Patients may present with fever, chills, and/or retrosternal pain. While it can affect any age, it is more common in young adults and can have 25–50% mortality [9, 10]. Radiographs show mediastinal widening, mediastinal air-fluid levels, and pneumomediastinum. CT findings may include diffuse fat stranding of the mediastinum, as well as organized fluid collections in the setting of esophageal perforation (Fig. 10). A clue to localizing the site of esophageal perforation is to look for extraluminal air which will typically be near the area of perforation. It is important to note that the findings in the post-surgical mediastinum can have a similar appearance to mediastinitis in the first 2–3 weeks. Esophagram with water-soluble contrast is recommended in patients with suspected esophageal perforation to better define the area of leak.

## Neurologic

The spectrum and symptoms from neurologic pathology of the thoracic inlet can be highly variable as many important nerves traverse this region, including the brachial plexus, vagus nerve, recurrent laryngeal nerves, phrenic nerve, and sympathetic chain. Some examples of non-neoplastic pathology are nerve sheath cysts, laryngeal nerve injury, brachial plexopathy, and post-radiation injury.

### Traction injury to the brachial plexus

**Fig. 11:**
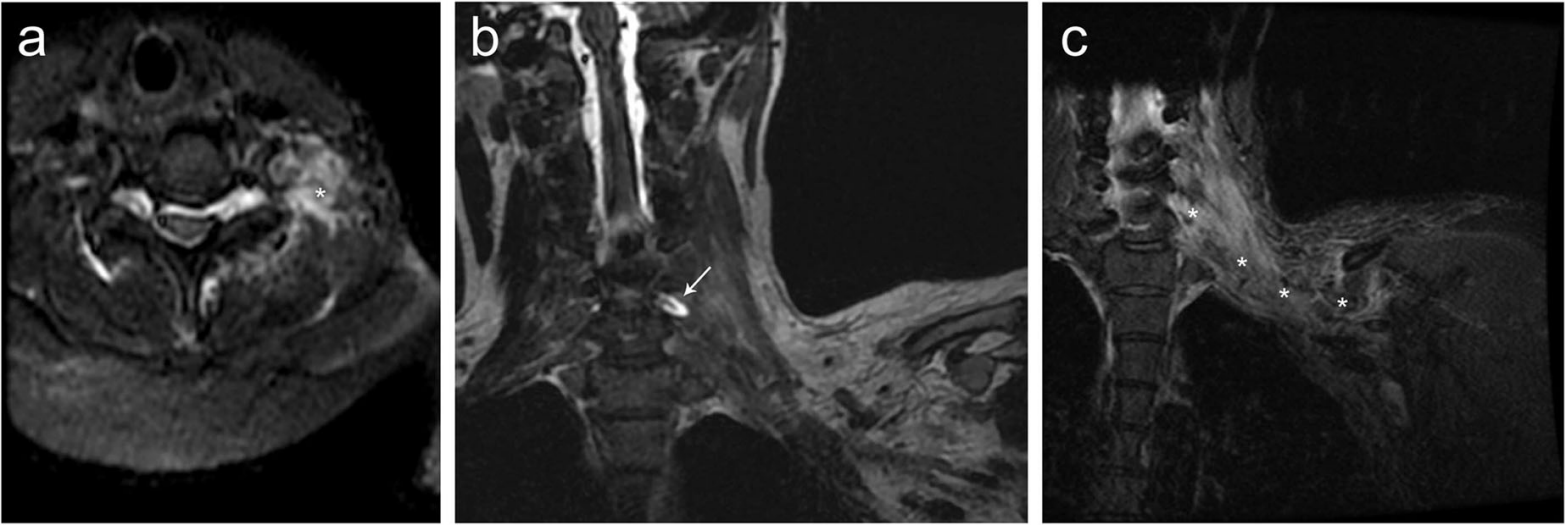
Traction injury of the brachial plexus. Patient with acute traumatic left C7 nerve root avulsion secondary to traction injury of the brachial plexus. Axial (**a**) short tau inversion recover (STIR) MR images demonstrate increased signal (asterisk) of the left neck base consistent with edema of the soft tissues at the site of injury. Coronal (**b**) STIR MR image demonstrate a bright signal fluid collection (arrow) which communicates with the cerebral spinal fluid of the spinal cord consistent with posttraumatic pseudomeningocele. Coronal (**c**) STIR MR image of a companion case demonstrating diffuse increased signal (asterisk) of the left neck base and thoracic inlet consistent with edema due to brachial plexus traction injury

In adults, brachial plexus avulsion or traction injury to the brachial plexus is typically related to high-energy trauma. In neonates, it is commonly due to excessive traction on the plexus by forceps during difficult delivery. Patients typically present with flail arm or incomplete paralysis. Diagnosis of brachial plexopathy is difficult on CT imaging; however, close evaluation may reveal pseudomeningocele or paraspinal hematoma [11]. MRI is the gold standard for diagnosing brachial plexopathy, typically demonstrating enlarged, hypo- to isointense neural elements on T1-weighted imaging (T1WI) and hyperintense on T2-weighted imaging (T2WI) due to stretch injury (Fig. 11). When there is an avulsion of a brachial plexus nerve root, a pseudomeningocele forms characterized by CSF hyperintensity within an empty root dilated root sleeve. Fat-saturated images are helpful in confirming edema and denervation changes, particularly of the cervical paraspinal muscles [11]. Myelogram may demonstrate a cerebral spinal fluid (CSF) leak and/or pseudomeningocele.

### Perineural cyst of the brachial plexus

**Fig. 12:**
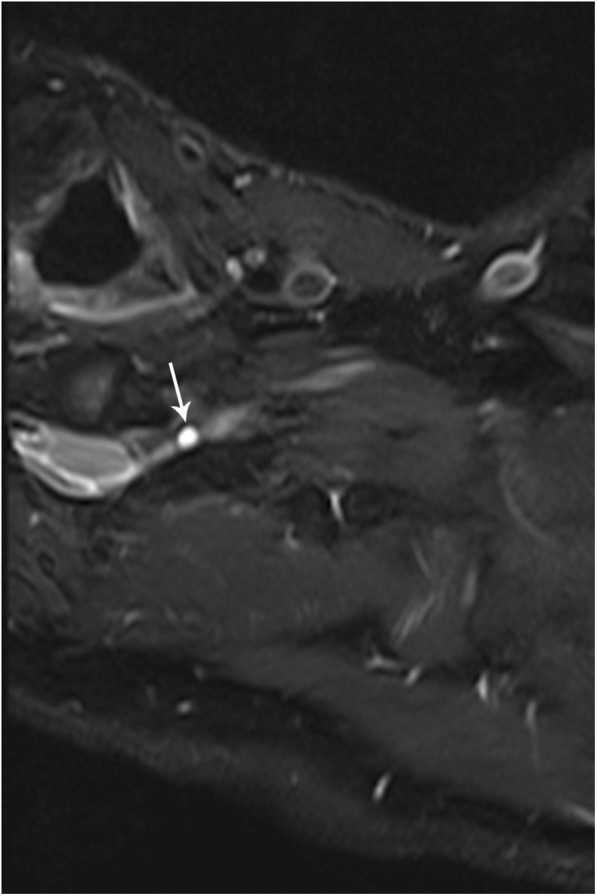
Perineural (Tarlov) cyst of the brachial plexus. Axial MR STIR image demonstrates a cerebral spinal fluid intensity simple cystic structure (arrow) arising from the left C6–7 nerve root sheath consistent with a perineural (Tarlov) cyst

Perineural cysts, also known as Tarlov cysts, are benign, typically asymptomatic, cerebrospinal fluid filled cystic cavities in the perineural recesses along the dorsal spinal nerve roots [12]. They are most often found along the sacral spine, however can also occur along the brachial plexus. Symptoms arise when a perineural cyst compresses a nerve root, presenting with symptoms of brachial plexopathy or radiculopathy, and these cysts are an important consideration as a possible etiology for brachial plexopathy when imaging exams are otherwise negative [12]. Perineural cysts are best seen on MR imaging and will be low signal on T1WI and high signal on T2WI (Fig. 12).

## Degenerative

Degeneration is a gradual deterioration of specific tissues, cells, or organs with impairment or loss of function due to injury, disease, or aging. This process occurs in all organ systems and in the thoracic inlet; this condition is most evident in the musculoskeletal, vascular, and digestive systems.

### Esophageal diverticulum

**Fig. 13:**
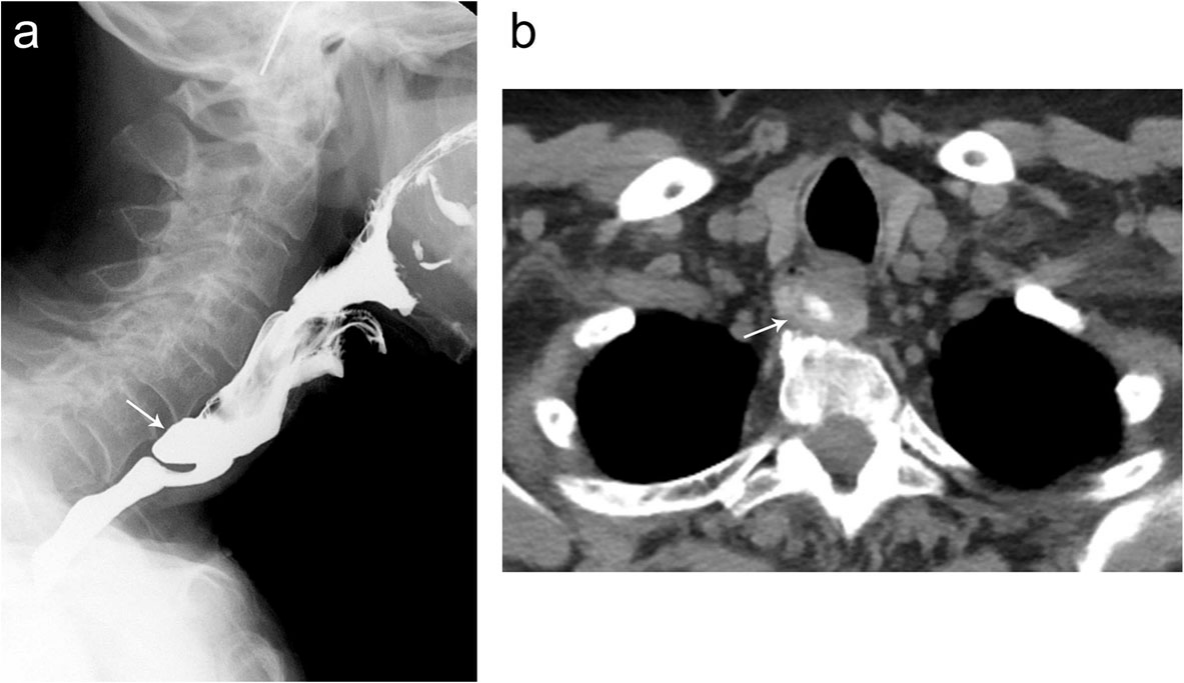
Zenker diverticulum. Lateral (**a**) fluoroscopic images demonstrate a focal outpouching from the posterior aspect of the esophagus containing an air-contrast level consistent with a Zenker diverticulum. Axial (**b**) noncontrast CT images from the same patient demonstrate residual oral contrast (arrow) within the Zenker diverticulum arising from the posterolateral aspect of the esophagus

**Fig. 14:**
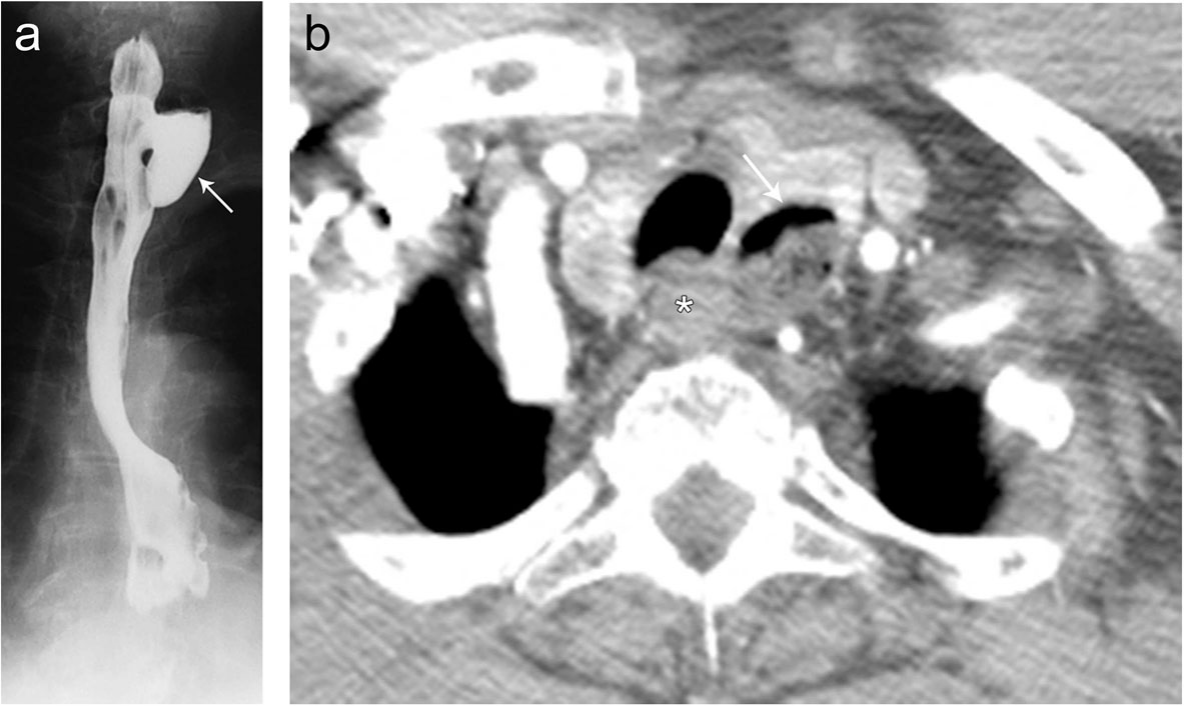
Killian-Jamieson diverticulum. Frontal (**a**) fluoroscopic images from an esophagram demonstrate a focal outpouching of the anterolateral aspect of the esophagus containing an air-fluid level consistent with a Killian-Jamieson diverticulum. These lesions are rarely bilateral and are typically smaller than a Zenker diverticulum (< 1.5 cm). Coronal (**b**) CT image from the same patient demonstrates air and oral contents (arrow) within the Killian-Jamieson diverticulum arising from the anterolateral aspect of the esophagus (asterisk)

Esophageal diverticula are saccular protrusions or outpouchings of the esophagus, classified according to their direction of protrusion and location. Zenker’s diverticula extend inferiorly behind the esophagus, just proximal to the upper esophageal sphincter (Fig. 13). Killian-Jamieson diverticula, conversely, are anterolateral protrusions into the Killian-Jamieson space, area of weakness just below the cricopharyngeal muscle (Fig. 14) [13]. These outpouchings can be unilateral or bilateral and are typically smaller than Zenker’s diverticulum, ranging from 3 to 20 mm. In both cases, patients typically present with dysphagia, regurgitation, and aspiration of undigested food, halitosis, hoarseness, and neck mass [13]. Imaging can demonstrate an air-fluid level or contrast-filled outpouching in the thoracic inlet [13].

### Tracheal diverticulum

**Fig. 15:**
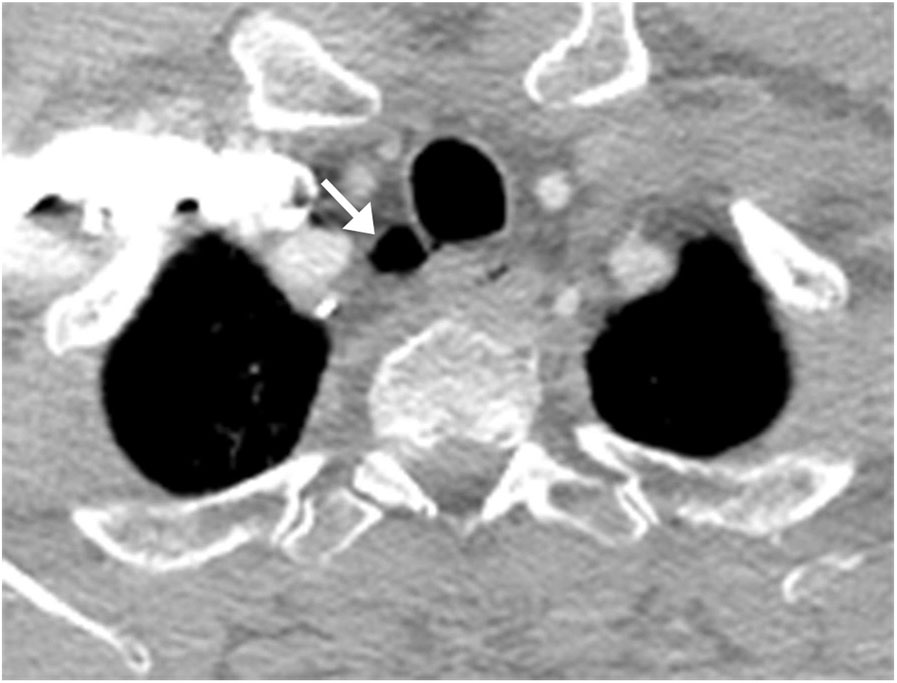
Tracheal diverticulum. Axial CT image demonstrates a focal outpouching (arrow) of the posterior right tracheal wall containing gas, consistent with a tracheal diverticulum

Tracheal diverticula can be congenital or acquired, with the congenital variant being less common. Congenital diverticula are often asymptomatic and incidentally detected unless associated with other conditions such as recurrent tracheobronchial infection. The distinction between the two entities is made pathologically, with the congenital tracheal diverticula composed of respiratory epithelium, smooth muscles, and cartilage, and the acquired tracheal diverticula are formed from respiratory epithelium only and lack smooth muscle and cartilage [14]. They both are characterized by single or multiple outpouchings of the tracheal wall, most commonly arising along the right lateral aspect of the trachea where the cartilage rings are deficient and there is no esophagus to support the adjacent tissue (Fig. 15). Most cases are asymptomatic and do not require treatment; however, these structures have the potential to become infected and form an abscess.

### Cervical osteophytosis

**Fig. 16:**
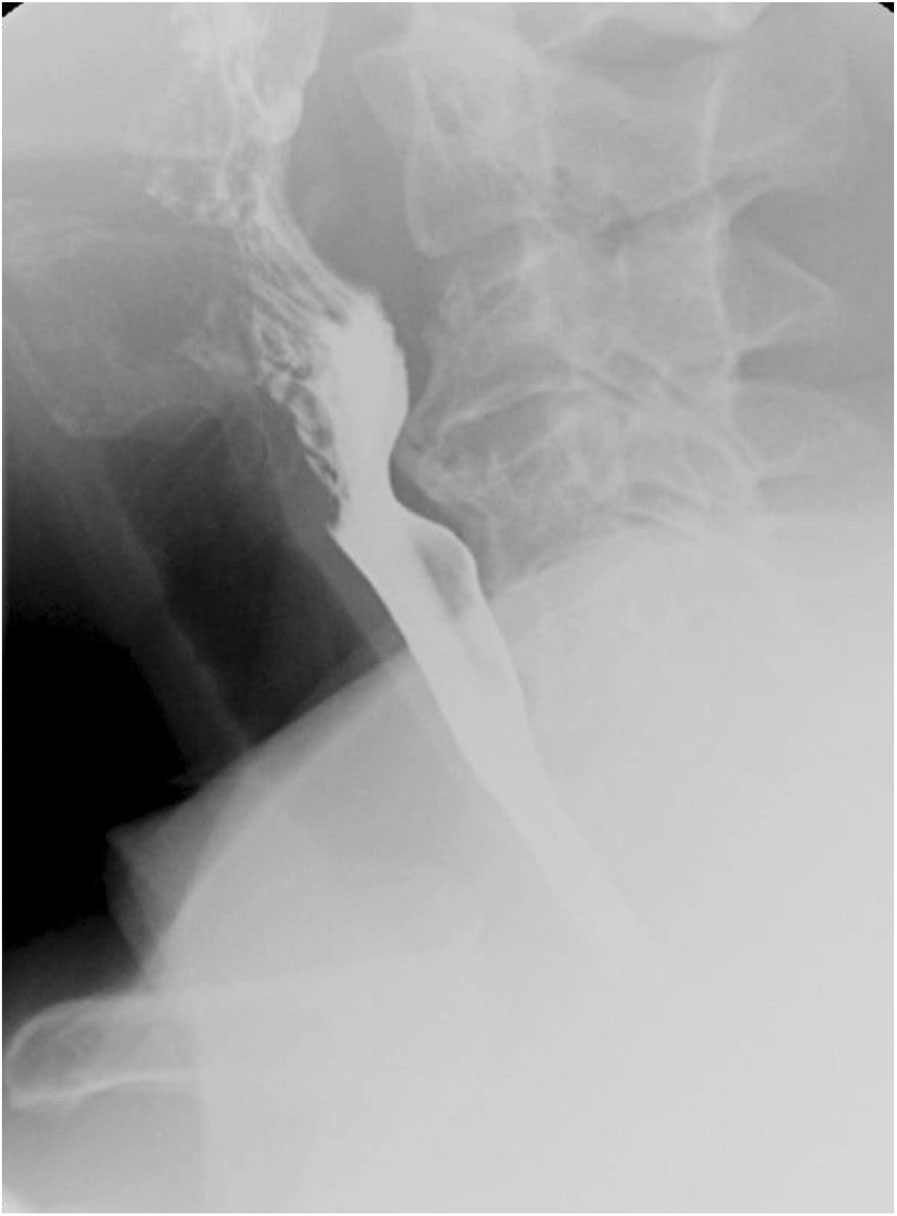
Cervical osteophytes. Lateral fluoroscopic view of the cervical spine during an esophagram demonstrating large anterior cervical osteophytes of the C3/4 level causing posterior impression on the esophagus during swallowing

Osteophytes are thorn-like or claw-like projections from the margins of vertebral endplates and are typically associated with spondylotic degenerative changes, findings which can be clearly seen on plain film and CT imaging (Fig. 16). Large cervical osteophytes can project anteriorly within the thoracic inlet and cause symptoms of dysphagia; therefore, it is an important imaging finding to include in reports when performing esophograms for dysphagia.

### Diffuse idiopathic skeletal hyperostosis

**Fig. 17:**
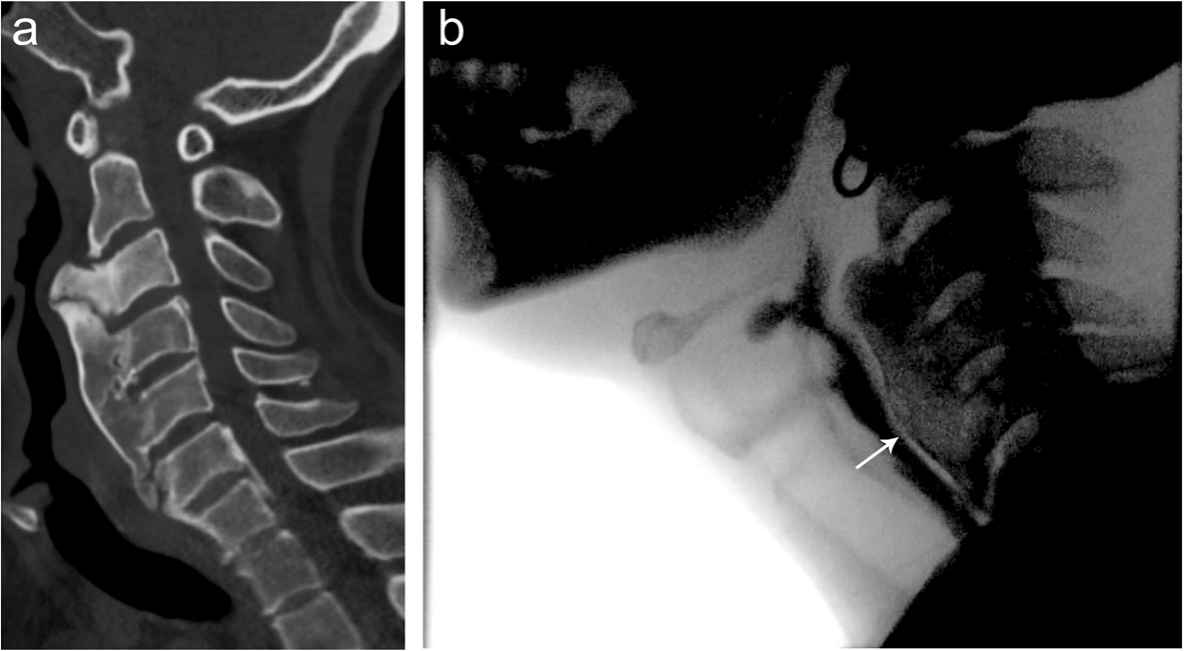
Diffuse idiopathic skeletal hyperostosis. Sagittal (**a**) noncontrast CT image of the cervical spine demonstrating large anteriorly flowing osteophytes extending from the C3–C7 levels without significant disc height loss consistent with the diagnosis of diffuse idiopathic skeletal hyperostosis (DISH). Lateral (**b**) fluoroscopy during a pharyngogram demonstrates posterior compression (arrow) of the cervical esophagus during swallowing

Diffuse idiopathic skeletal hyperostosis (DISH) is a distinct entity from cervical osteophytosis, is of unknown etiology, and causes flowing anterior vertebral ossification. Primary diagnostic criteria include flowing anterior ossification extending over at least four contiguous vertebral bodies, no sacroiliac or apophyseal ankylosis, mild degenerative disc changes, and no facet ankyloses (Fig. 17). DISH can coexist with spondylosis, however complicating the diagnosis. DISH may cause dysphagia due to a combination of mechanical compression and inflammation/fibrosis of the esophageal wall. Associated adjacent ligament ossification around the cervical spine may also result in pain and/or dysphagia. Complications may include decreased mobility due to anterior longitudinal ligament fracture following minor trauma in long column fusion and osteoporosis [15]. CT imaging can confirm the location of ossification, as well as subtle transverse fractures of the anterior longitudinal ligament following minor trauma (Fig. 17). On MR imaging, flowing anterior longitudinal ligament ossification is hypointense on T1W1 and T2WI, unless there is substantial fatty marrow content, in which case they may be hyperintense [16].

## Iatrogenic

Iatrogenic complications are a broad category of conditions that directly result from healthcare treatment or intervention. Common causes at the level of the thoracic inlet include complications following surgical procedures and post-radiation changes leading to fibrosis and scarring of the region.

### Tracheoesophageal fistula

**Fig. 18:**
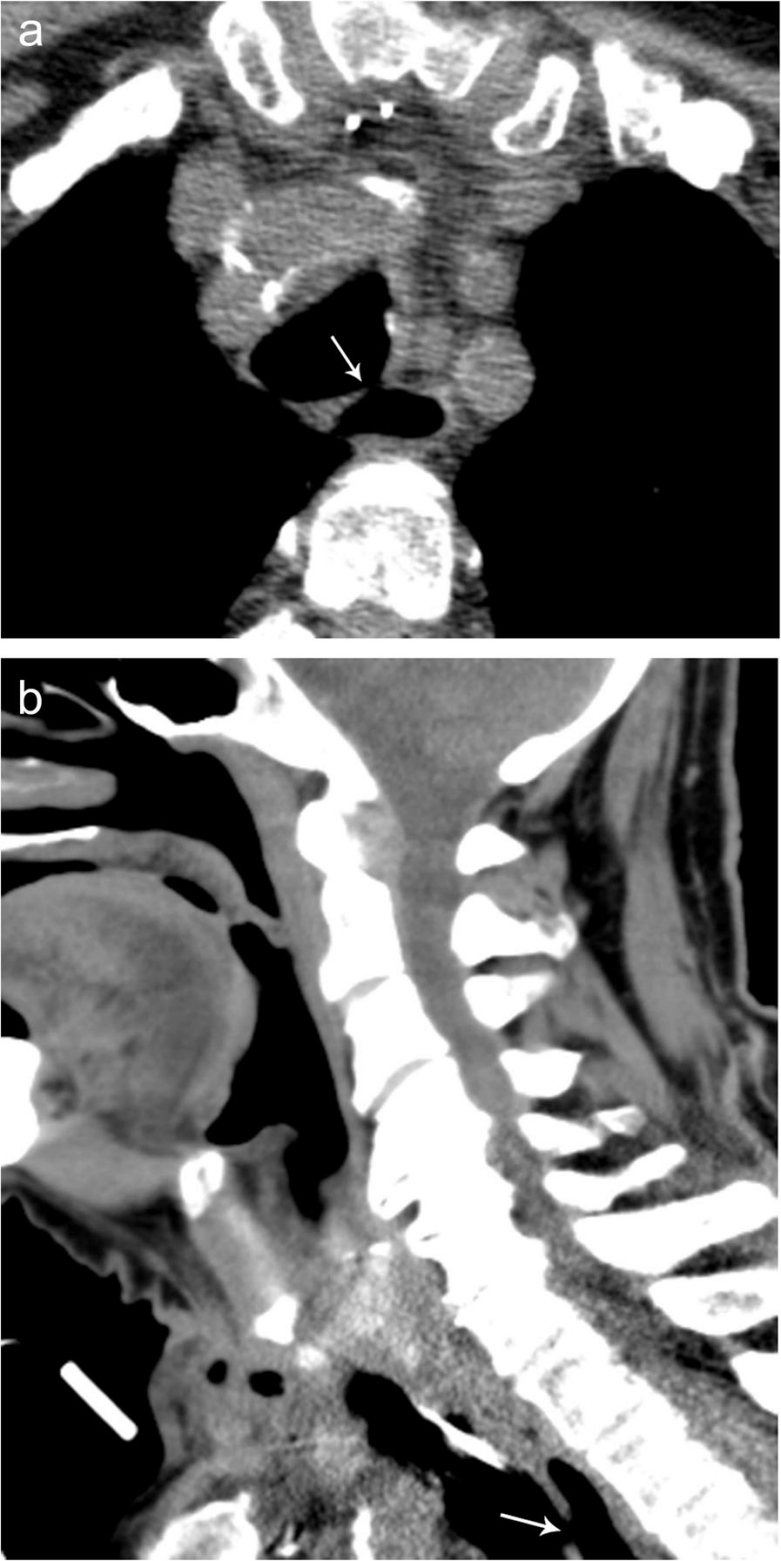
Tracheoesophageal fistula. Axial (**a**) and sagittal (**b**) noncontrast CT images demonstrating a communication (arrow) between the trachea and esophagus consistent with a tracheoesophageal fistula as a complication of esophageal stent placement in a patient with a history of esophageal trauma

While tracheoesophageal fistula (TEF) is almost always congenital in the pediatric population, in adults, it can occur iatrogenically as a complication of malignancy, malignancy treatment, or surgical intervention (Fig. 18). Other less common causes of TEF include sequelae of infection and inflammation as can occur in post-foreign body impaction. Symptoms include excessive oral secretions, cyanosis, choking, and coughing. Fluoroscopic imaging is often the best imaging modality for diagnosis allowing for a real time and dynamic look of the esophageal lumen and may identify the site of communication. Unfortunately, CT and MR imaging often do not visualize the defect if the esophagus is collapsed. Administering oral contrast immediately before CT imaging can improve the detection of the fistulous track connecting the esophagus to the trachea. CT imaging can also be used to evaluate for complications of TEF including evidence of aspiration within the lung parenchyma.

## Congenital

Some frequently encountered congenital anomalies of the thoracic inlet are tracheobronchomegaly, dilated thoracic duct, branchial cleft cysts, narrowed thoracic inlet, and fibromatosis colli, as well as the previously discussed tracheoesophageal fistula and vascular anomalies including the aberrant right subclavian artery and partial anomalous pulmonary venous return.

### Congenital tracheobronchomegaly

**Fig. 19:**
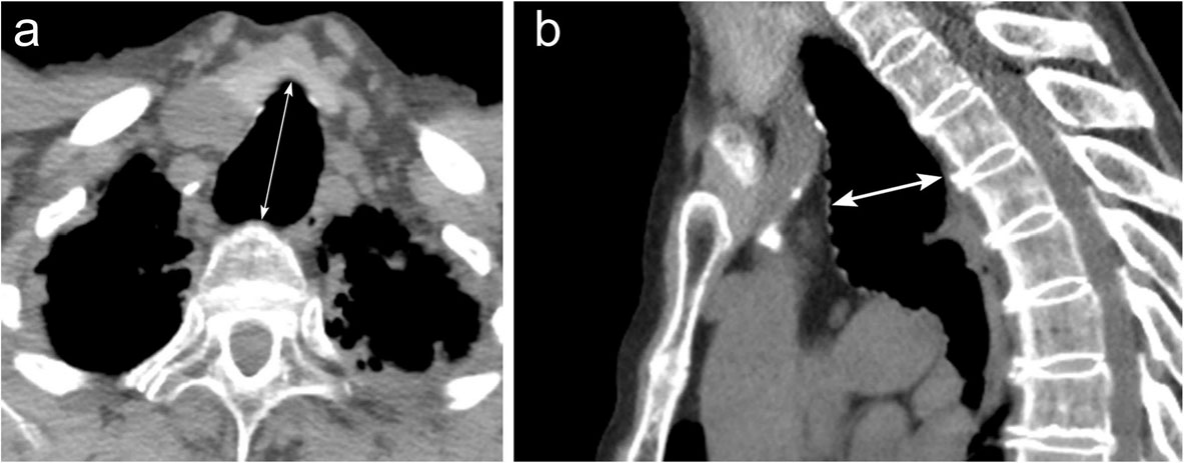
Tracheobronchomegaly. Axial (**a**) and sagittal (**b**) noncontrast CT images of the thoracic inlet demonstrating dilatation of the trachea, measuring > 3 cm as can be seen in the setting of tracheobronchomegaly

Tracheobronchomegaly, also known as Mounier-Kuhn syndrome, can be either congenital or acquired. In congenital patients, this condition develops secondary to an underlying abnormality involving the connective tissues, such as Ehlers-Danlos syndrome or cutis laxa, causing atrophy of the tracheal and mainstem bronchial elastic tissue and smooth muscle leading to significant central airway dilatation [17]. Patients typically present with cough and dyspnea, which may mimic or coexist with chronic obstructive pulmonary disease. Radiographs may demonstrate marked dilatation of the trachea and central bronchi. On CT imaging, the condition should be suspected if the trachea, right main bronchus, and left main bronchus measure greater than 3 cm, 2.0–2.4 cm, and 1.5–2.3 cm respectively, with the trachea measurement commonly performed approximately 2 cm above the level of the aortic arch [17] (Fig. 19). Other CT findings may include posterior tracheobronchial diverticulosis, bronchiectasis, and tracheobronchial collapse with expiration imaging.

### Dilated thoracic duct

**Fig. 20:**
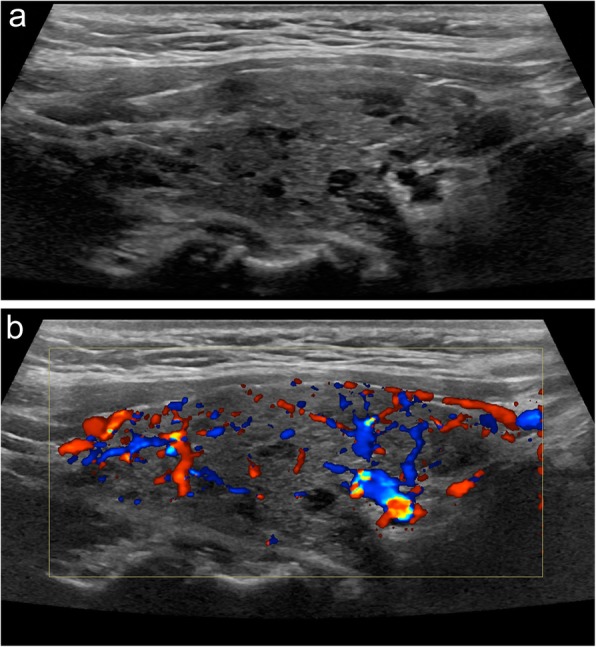
Dilated thoracic duct. Axial (**a**) and coronal (**b**) contrast CT images demonstrating dilated thoracic duct (arrow) draining into the confluence of the left subclavian and left internal jugular veins. This is considered a normal variant and is important to not mistake it for a pathologic lesion

The thoracic duct drains lymph from the lower extremities, abdomen, and left chest. This structure crosses the thoracic inlet posterior to the left subclavian artery, anterior to the anterior scalene muscle/phrenic nerve, and empties into the venous system at the confluence of the left subclavian and left internal jugular veins in the carotid space [18]. The thoracic duct may be identified in about 55% of cases on routine neck CT imaging [19]. The average diameter of a thoracic duct is 4.8 mm [19]. This structure, however, may enlarge after a large fatty meal, and dilatation of the structure should be considered a normal anatomic variant and considered an incidental finding if seen on imaging. CT imaging will show a left-sided non-enhancing tubular structure which drains into the confluence of the left subclavian and left internal jugular veins [1] (Fig. 20). On MR imaging, the thoracic duct has hyperintensity on T2-weighted imaging, similar to cerebral spinal fluid (CSF) [1, 19].

## Autoimmune

Autoimmune conditions are generally systemic, with some demonstrating propensity for specific organs, including those traversing the thoracic inlet. For example, systemic sclerosis can affect the esophagus causing esophageal dilatation [20]. Autoimmune thyroiditis such as Hashimoto’s thyroiditis causes thyroid enlargement with nodules and can have an association with lymphoma [21]. Antiphospholipid syndrome is associated with venous and arterial thrombosis [22].

### Hashimoto’s thyroiditis

**Fig. 21:**
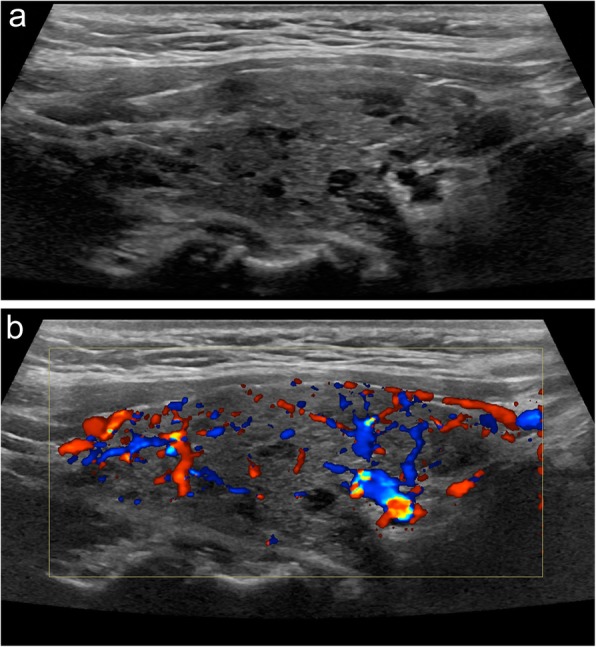
Hashimoto’s thyroiditis. Grayscale (**a**) ultrasound imaging demonstrating diffuse enlargement of the thyroid gland with heterogeneous parenchyma and micronodular echotexture. Color Doppler (**b**) ultrasound imaging demonstrates diffuse hypervascularity, reflecting the early phase of disease secondary to the hypertrophic action of thyroid-stimulating hormone (TSH)

Hashimoto’s thyroiditis, also known as chronic autoimmune thyroiditis or chronic lymphocytic thyroiditis, is the most common cause of thyroiditis and hypothyroidism. Patients typically present with gradual painless enlargement of the thyroid gland and symptoms related to hypothyroidism. Grayscale ultrasound tends to vary with different stages of the disease and extent of involvement. The thyroid is typically enlarged with heterogeneous echotexture. Multiple, discrete hypoechoic micronodules from 1 to 6 mm in diameter are strongly suggestive of chronic thyroiditis (Fig. 21). On color Doppler, the thyroid may demonstrate normal or decreased blood flow in later stages; however, it may occasionally be hypervascular, reflecting early phase of the disease and hypertrophic action of thyroid-stimulating hormone (Fig. 21). CT shows symmetric enlargement of thyroid with diffuse decreased density [21].

### Thymic hyperplasia

The thymus has a variable appearance, typically soft tissue density at birth through adolescence, reaching a maximal size by the late teens, then gradually becoming fatty replaced through adulthood. The gland can extend from the lower border of the thoracic inlet through the fourth costal cartilage anteriorly.

**Fig. 22:**
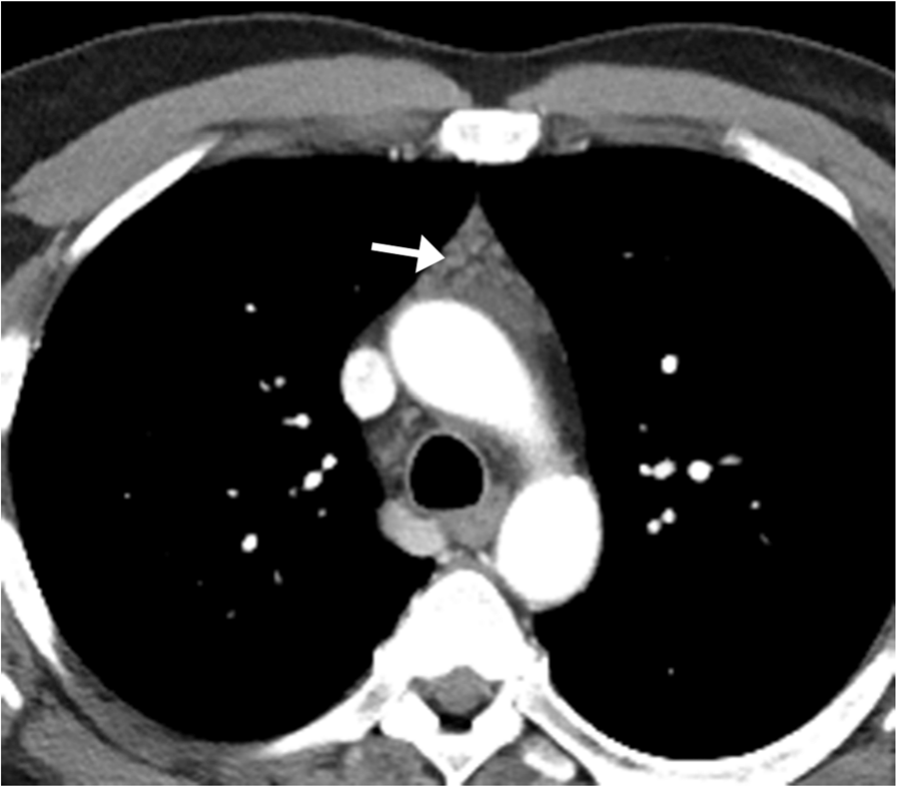
Thymic hyperplasia. Axial CT image of a 60-year-old patient undergoing chemotherapy demonstrates an anterior mediastinal mass (arrow) with the expected location, configuration, and attenuation of the thymus gland, consistent with rebound thymic hyperplasia secondary to chemotherapy. It is important to distinguish thymic hyperplasia from thymoma, which can present as asymmetry of the lobes, focal contour abnormality, and demonstrate focal increased FDG uptake

Thymic hyperplasia is characterized by an increase in volume of the thymus by more than 50% from baseline. The enlarged thymus can extend superiorly to the level of the thoracic inlet and into the retrovisceral space. Thymic hyperplasia can occur in many conditions, including hyperthyroidism, acromegaly, Addison’s disease, stress, chemotherapy, radiotherapy, and myasthenia gravis. The normal average thymic thickness on an inspiratory phase CT study is 1.1 cm between ages 6 and 19 years and 0.8 cm in adults older than 20 years, and an increased thickness of the thymus greater than 1.3 cm beyond age 20 should raise the suspicion for thymic hyperplasia (Fig. 22) [23]. A hyperplastic thymus typically demonstrates a normal shape, with homogenous attenuation and signal. On MR imaging, a signal drop on the opposed-phase imaging compared to in-phase imaging indicates a normal thymus or thymic hyperplasia is present as the thymus normally contains interspersed fat [24]. It is important to distinguish thymic hyperplasia from thymoma, which can present as asymmetry of the lobes, focal contour abnormality, or as a discrete soft tissue mass. Thymic hyperplasia demonstrates homogenous low-grade fluorodeoxyglucose (FGD) uptake, whereas thymoma has more focal FDG uptake.

## Traumatic

There are many causes of traumatic injury to the thoracic inlet, each with unique features depending on the method of trauma and the structures involved. Importantly, trauma to the neck accounts for 5–10% of all serious traumatic injuries [25]. The two most common forms of trauma are blunt force trauma and penetrating trauma.

### Pneumomediastinum

**Fig. 23:**
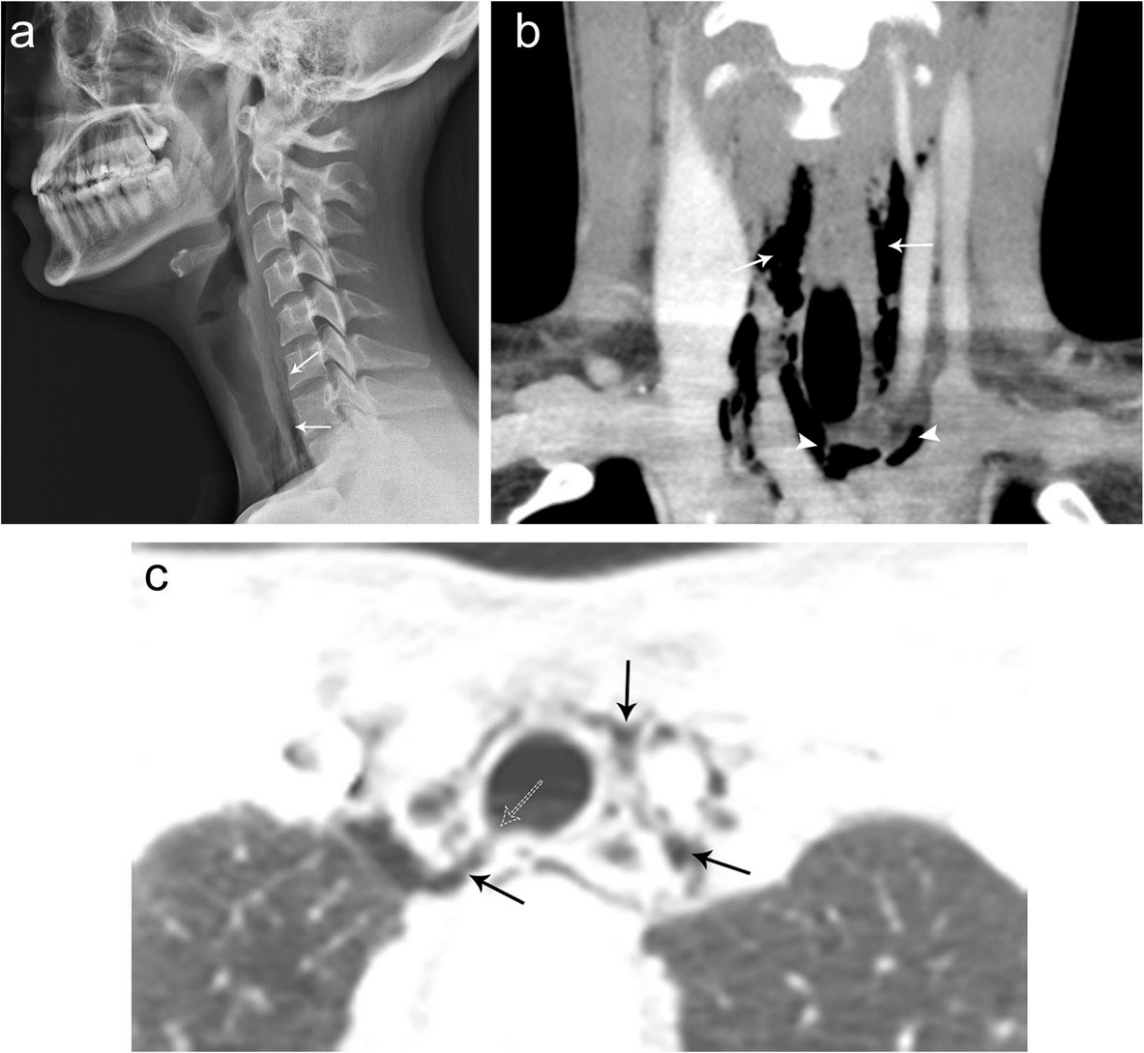
Subcutaneous emphysema and pneumomediastinum. Lateral cervical spine radiograph (**a**) showing linear streaks of air (arrows) tracking along the prevertebral soft tissues of the thoracic inlet consistent with subcutaneous emphysema. Coronal (**b**) contrast CT image demonstrates subcutaneous air (arrows) tracking along the cervical soft tissues and fascial planes extending into the superior mediastinum (arrowhead). Axial CT chest image in lung (**c**) windows demonstrating air (solid arrows) within fascial planes of the superior mediastinum. Note the tracheal defect (dashed arrow) which was the source of the pneumomediastinum and subcutaneous emphysema on the lung window images

Pneumomediastinum most commonly results from traumatic rupture of the trachea or esophagus, which occurs in 10% of blunt chest trauma cases, or as a result of spontaneous alveolar rupture with air dissecting from the pulmonary interstitium (Macklin effect) [26]. Pneumomediastinum may then extend into the neck and shoulder soft tissues. Alternatively, subcutaneous air from penetrating neck injuries can dissect inferiorly causing pneumomediastinum. A careful inspection of the organs traversing the thoracic inlet is critical in penetrating injuries involving the neck and/or chest. Positive findings in one region may trigger further investigation of the other region and may help identify the etiology of pneumomediastinum. Patients with pneumomediastinum commonly present with chest/neck pain, cough, and dyspnea [27]. On radiographs, linear lucencies surrounding the mediastinal structures indicate the presence of air, often more conspicuous on lateral radiographs. CT imaging is much more sensitive for the detection of air within the mediastinal structures and can often help to identify the cause of pneumomediastinum and look for additional sites of injury [27] (Fig. 23).

### Vascular injury

**Fig. 24:**
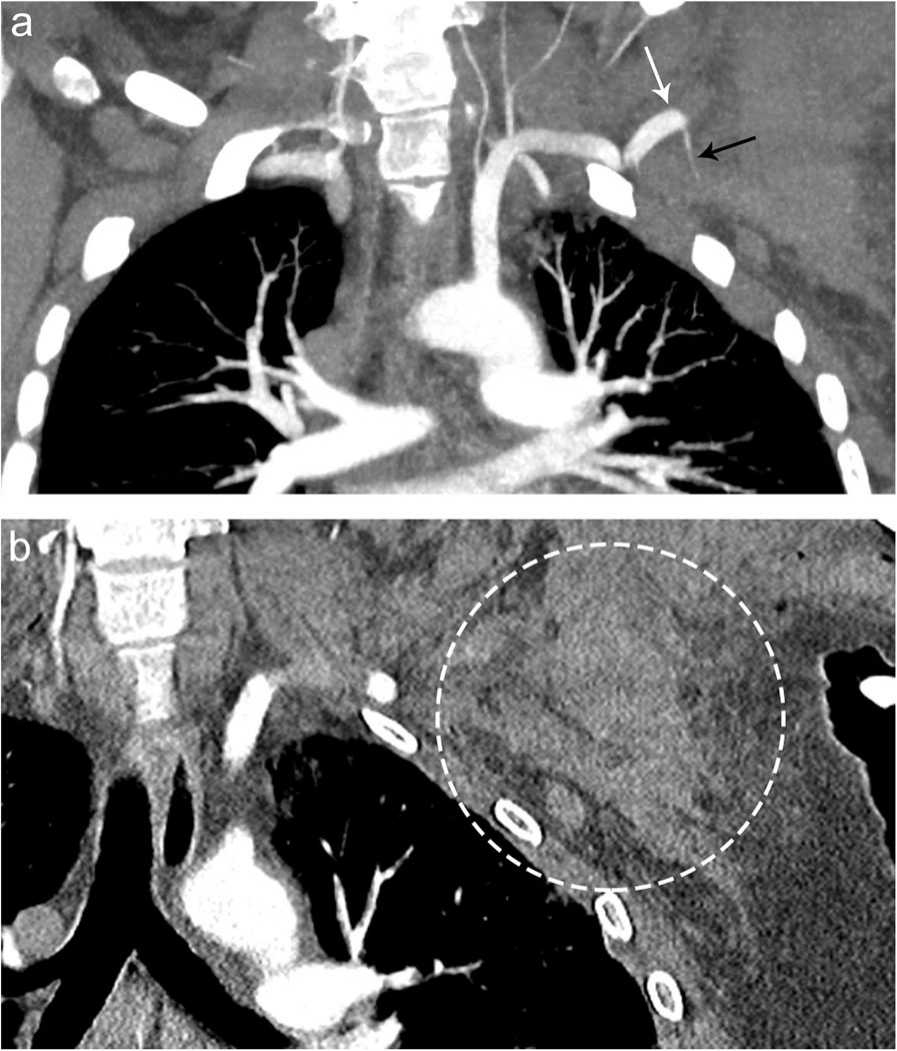
Transection of the left axillary artery. Patient presenting with multiple traumatic fractures and a cold left arm. Coronal (**a**) contrast CT image demonstrates abrupt cutoff of the left axillary artery (white arrow), along with active extravasation of contrast material (black arrow). Coronal (**b**) contrasted CT images demonstrate associated hematoma (dashed circle) in this corresponding region

Vascular injuries are commonly encountered in trauma, with cervical vascular injuries present in 0.1–1.1% of all trauma cases. Common vascular injuries include transection, dissection, pseudoaneurysm, and/or thrombosis. Patients can present with ipsilateral pain to the affected side, bruit, and neurologic symptoms due to ischemia [28]. CT angiogram in the arterial phase is the preferred modality to diagnose vascular pathology and look for signs of active extravasation. Signs of arterial injury include active extravasation, pseudo-aneurysm formation, abrupt narrowing of an artery, loss of opacification of an arterial segment, or arteriovenous fistula formation (Fig. 24).

### Sternocleidomastoid muscle hematoma

**Fig. 25:**
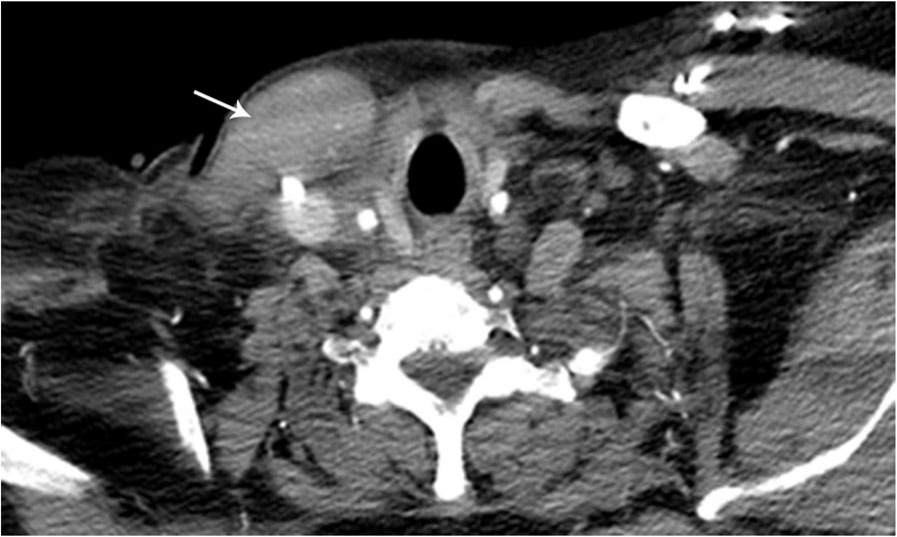
Right sternocleidomastoid hematoma. Axial contrast CT images demonstrate an asymmetric enlargement of the right sternocleidomastoid muscle (arrow) in a trauma patient consistent with an intramuscular hematoma

Sternocleidomastoid muscle hematoma can be seen in the setting of blunt neck trauma. Large hematomas can compress the critical vascular and respiratory structures along the neck/thoracic inlet and can therefore quickly become life-threatening. Physical examination may be misleading as this condition may be mistaken for a thyroid goiter. Patients can present with varying degrees of clinical symptoms, including soft tissue swelling and pain, vital sign instability, hypotension, hypoxia, and possible respiratory arrest from compression of the trachea by the hematoma [25]. Imaging will demonstrate asymmetric enlargement of the injured muscle with possible identification of a fluid collection and/or active extravasation of contrast within the muscle (Fig. 25). It is important to report this finding as it may necessitate intubation or neck dissection for airway protection and decompression respectively.

## Endocrine

Endocrinological organs to consider in the region of the thoracic inlet include the parathyroid and thyroid glands. The thyroid isthmus lies just above the level of the thoracic inlet in the midline. The parathyroid glands are normally paired bilaterally, typically located posterior to the thyroid gland. However, their location can be variable, occurring anywhere along the thyrothymic tract, extending from the angle of the mandible to the lower aspect of the superior mediastinum [29]. Abnormalities and variants of these organs are commonly reported, including thyroid nodules, thyroid pyramid, and parathyroid hyperplasia. Thyroid nodules are incidental findings in 4–8% of adults [30, 31].

### Thyroid goiter

**Fig. 26:**
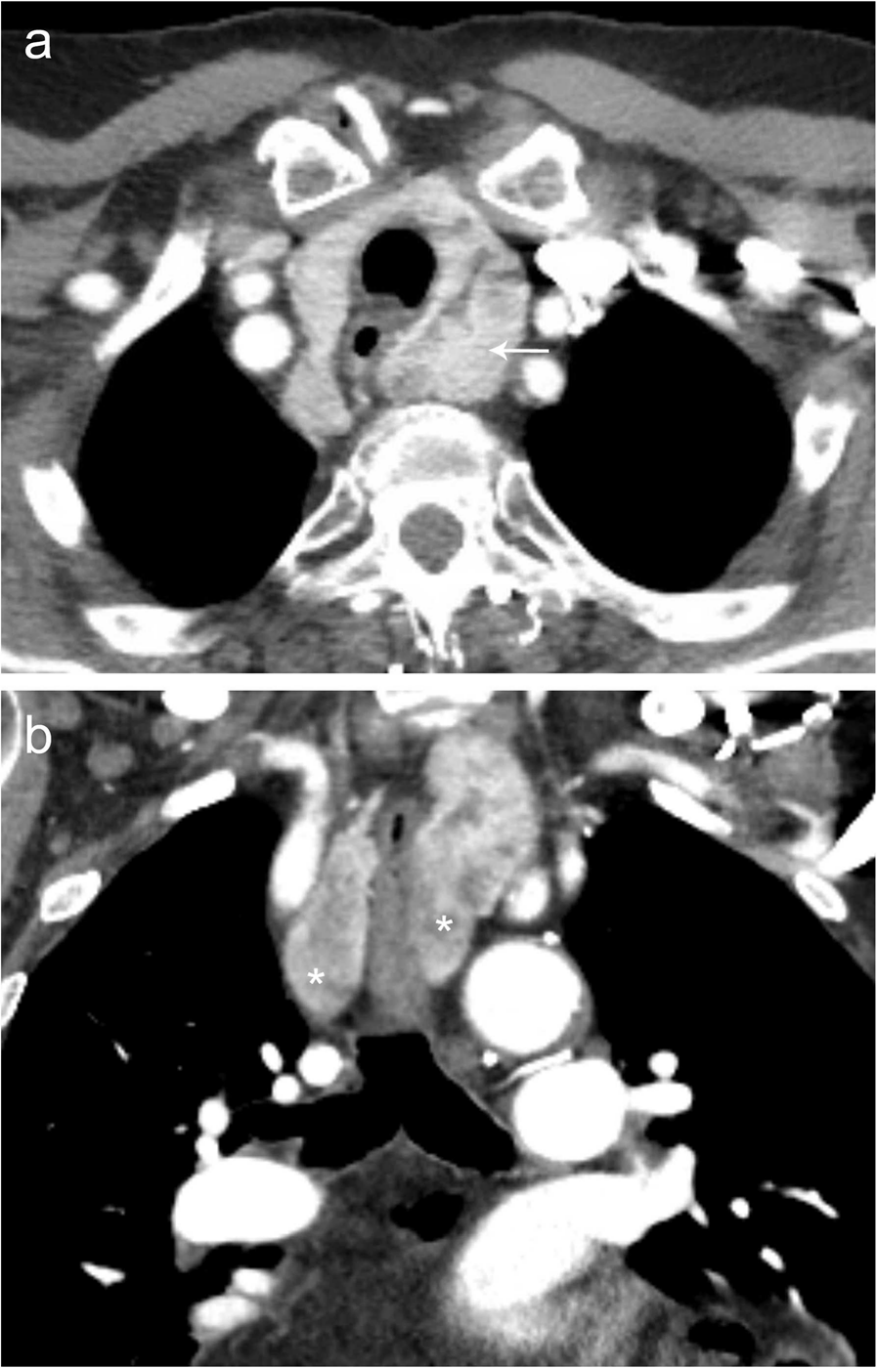
Multinodular thyroid goiter. Axial (**a**) and coronal (**b**) contrasted CT imaging demonstrates an enlarged heterogeneous multinodular appearance of the thyroid gland. Note that this enlargement is causing rightward deviation of the trachea (arrow) and substernal extension (asterisk) into the anterior mediastinum

Thyroid goiter is a condition in which there is enlargement of the thyroid gland due to multiple nodules with increased, decreased, or normal function. Thyroid goiters can be defined according to their location and origin: cervical goiter, retrosternal/substernal goiter, mediastinal goiter, primary goiter (origin in ectopic thyroid tissue), and secondary goiter (origin in cervical thyroid). Patients typically are asymptomatic, but may present with dyspnea and wheezing if the trachea is compressed (Fig. 26). Surgical excision is the definitive therapy for thyroid goiter. However, many do not have compressive symptoms and therefore do not require treatment. Observation is considered for small lesions and elderly patients. Radiographs may demonstrate neck base/superior mediastinal mass with or without intrinsic calcification, tracheal displacement, and tracheal narrowing. CT can help delineate the mass, which may have cystic changes, high attenuation (70–80 HU) due to intrinsic iodine and calcification. There can be prolonged and sustained enhancement after IV contrast [32].

### Madelung disease

**Fig. 27:**
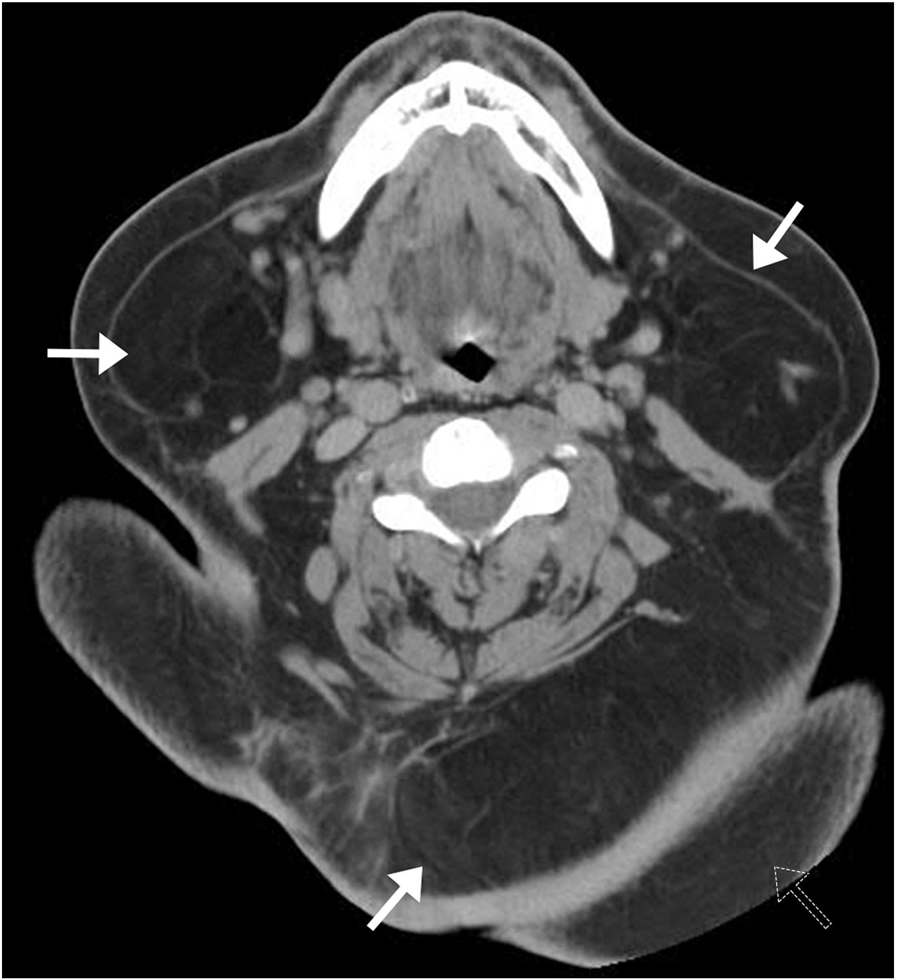
Madelung disease. Axial CT image of a patient with history of alcoholism presenting with shortness of breath demonstrates diffuse symmetric unencapsulated fatty accumulation of the neck (arrows) and upper back (dashed arrow). In the given clinical context, this is most consistent with Madelung disease or benign symmetric lipomatosis

Madelung disease, also known as benign symmetric lipomatosis, is a rare disease of lipodystrophy characterized by diffuse, symmetric, unencapsulated fatty accumulation most commonly in the neck, upper back, shoulder girdle, and upper extremities (Fig. 27). Fat accumulation is also seen in the deep spaces, including the deep layer of sternocleidomastoid muscles, trapezius muscles, periphery of paravertebral muscles, and surrounding the salivary glands. This process is characterized by abnormal fatty tissue metabolism causing accelerated fatty tissue proliferation. It has been associated with alcoholism and in middle-aged men of Mediterranean descent. Often asymptomatic, however, long-term lipomatous deposits can be cosmetically deforming and may compress the aerodigestive tract and great veins, causing dysphagia, dyspnea, or venous stasis [33]. The diagnosis is typically made based on history, clinical appearance, and imaging. The definitive treatment is surgical therapy. Both CT and MRI can accurately identify the distribution and involvement of the disease, along with its sequelae including compression of surrounding organs. These imaging modalities play important roles in distinguishing Madelung disease from obesity (more diffuse process), lipoma (encapsulated lesions), and other soft tissue tumors, as well as aiding surgical planning.

## Conclusion

As seen, the thoracic inlet is a critical landmark in radiology, as it is a central conduit for many organ systems, and thus allowing itself to a vast array of non-neoplastic pathology. It is therefore important to critically evaluate this region and be aware that it may be overlooked on imaging of the neck or chest. The thoracic inlet marks a critical junction between the neck and chest imaging. A detailed examination of this region is essential when reviewing both neck and thoracic studies. The mnemonic VINDICATE is a helpful guide to methodically create differential diagnoses for non-neoplastic pathology encountered in this region.

## Data Availability

Not applicable.

## Abbreviations

CS: Carotid space
CT: Computed tomography
DCF: Deep cervical fascia
DISH: Diffuse idiopathic skeletal hyperostosis
DLDCF: Deep layer of the deep cervical fascia
DS: Danger space
FDG: Fluorodeoxyglucose
IJV: Internal jugular vein
MLDCF: Middle layer of the deep cervical fascia
MR: Magnetic resonance
PAPVR: Partial anomalous pulmonary venous return
PCS: Posterior cervical space
PVS: Perivertebral space
RPS: Retropharyngeal space
SCF: Superficial cervical fascia
SLDCF: Superficial layer of the deep cervical fascia
SVC: Superior vena cava
US: Ultrasound
VS: Visceral space
